# Barley has two peroxisomal ABC transporters with multiple functions in β-oxidation

**DOI:** 10.1093/jxb/eru243

**Published:** 2014-06-09

**Authors:** Guillermina M. Mendiondo, Anne Medhurst, Carlo W. van Roermund, Xuebin Zhang, Jean Devonshire, Duncan Scholefield, José Fernández, Barry Axcell, Luke Ramsay, Hans R. Waterham, Robbie Waugh, Frederica L. Theodoulou, Michael J. Holdsworth

**Affiliations:** ^1^Division of Plant and Crop Sciences, School of Biosciences, University of Nottingham, Sutton Bonington Campus, Loughborough LE12 5RD, UK; ^2^Laboratory of Genetic Metabolic Diseases, Academic Medical Centre, University of Amsterdam, 1105 AZ, Amsterdam, The Netherlands; ^3^Biological Chemistry and Crop Protection Department, Rothamsted Research, Harpenden AL5 2JQ, UK; ^4^Plant Biology and Crop Science Department, Rothamsted Research, Harpenden AL5 2JQ, UK; ^5^SABMiller plc., SABMiller House, Church Street, West Woking, Surrey GU21 6HS, UK; ^6^Division of Plant Sciences, College of life Sciences, University of Dundee and The James Hutton Institute, Invergowrie, Dundee DD2 5DA, UK

**Keywords:** Aleurone, gene duplication, germination, indole butyric acid, oil body, seed size.

## Abstract

In oilseed plants, peroxisomal β-oxidation functions not only in lipid catabolism but also in jasmonate biosynthesis and metabolism of pro-auxins. Subfamily D ATP-binding cassette (ABC) transporters mediate import of β-oxidation substrates into the peroxisome, and the *Arabidopsis* ABCD protein, COMATOSE (CTS), is essential for this function. Here, the roles of peroxisomal ABCD transporters were investigated in barley, where the main storage compound is starch. Barley has two *CTS* homologues, designated *HvABCD1* and *HvABCD2*, which are widely expressed and present in embryo and aleurone tissues during germination. Suppression of both genes in barley RNA interference (RNAi) lines indicated roles in metabolism of 2,4-dichlorophenoxybutyrate (2,4-DB) and indole butyric acid (IBA), jasmonate biosynthesis, and determination of grain size. Transformation of the *Arabidopsis cts-1* null mutant with *HvABCD1* and *HvABCD2* confirmed these findings. *HvABCD2* partially or completely complemented all tested phenotypes of *cts-1*. In contrast, *HvABCD1* failed to complement the germination and establishment phenotypes of *cts-1* but increased the sensitivity of hypocotyls to 100 μM IBA and partially complemented the seed size phenotype. *HvABCD1* also partially complemented the yeast *pxa1/pxa2Δ* mutant for fatty acid β-oxidation. It is concluded that the core biochemical functions of peroxisomal ABC transporters are largely conserved between oilseeds and cereals but that their physiological roles and importance may differ.

## Summary

Peroxisomal ABC transporters have been studied extensively in *Arabidopsis* but not in monocotyledonous species. Using barley, it is shown that their biochemical functions are conserved in flowering plants.

## Introduction

The peroxisome is the sole site of β-oxidation of fatty acids and related molecules in plants and fungi, and is required for very long chain fatty acid metabolism and signalling in mammals (Baker *et al.*, 2006; Poirier *et al.*, 2006; Wanders and Waterham, 2006). In plants, β-oxidation is important not only during germination, seedling establishment, fertilization, and dark-induced senescence, but also in a number of additional key roles (reviewed in Theodoulou and Eastmond, 2012; Linka and Theodoulou, 2013). These include biosynthesis of the hormones jasmonic acid (JA) and indole acetic acid (IAA), production of volatile benzenoids and benzoyloxyalkylglucosinolates, and also salicylic acid biosynthesis (Reumann *et al.*, 2004; Kliebenstein *et al.*, 2007; Gfeller *et al.*, 2010; Strader *et al.*, 2011; Klempien *et al.*, 2012; Lee *et al.*, 2012; Qualley *et al.*, 2012; Bussell *et al.*, 2014).

Transport of β-oxidation substrates into the peroxisome is mediated by ATP-binding cassette (ABC) proteins belonging to subfamily D (Theodoulou *et al.*, 2006). In mammals and fungi, peroxisomal ABCD transporters are expressed as ‘half-size’ proteins, each containing a transmembrane domain (TMD) and a nucleotide-binding domain (NBD), which homo- or heterodimerize to form a functional transporter (Theodoulou *et al.*, 2006). Plants are unusual in having ‘full-size’ peroxisomal ABCD transporters, in which two dissimilar domains are expressed as a single polypeptide with the structure TMD–NBD–TMD–NBD, which is considered to represent a fused heterodimer (Verrier *et al.*, 2008). The prototypical plant member of the ABCD subfamily is the *Arabidopsis thaliana* protein, COMATOSE (CTS; also known as AtPXA1, PED3, ACN2, AtABCD1; Zolman *et al.*, 2001; Footitt *et al.*, 2002; Hayashi *et al.*, 2002; Hooks *et al.*, 2007). Although the transporter is functional when the two halves are artificially expressed as separate proteins, both are required for function (Nyathi *et al.*, 2012). Biochemical and genetic evidence suggests that CTS accepts fatty-acyl CoAs as substrates and cleaves off the coenzyme A (CoA) moiety during the transport cycle (Footitt *et al.*, 2002; Nyathi *et al.*, 2010; De Marcos Lousa *et al.*, 2013). Acyl-activating enzymes then re-esterify the fatty acids to CoA in the peroxisome lumen, which is a prerequisite for entry into β-oxidation (Fulda *et al.*, 2004; De Marcos Lousa *et al.*, 2013). This unusual transport mechanism appears to be common to plant, yeast, and mammalian ABCD proteins (van Roermund *et al.*, 2012).

Alleles of *cts* have been identified in several forward genetic screens in *Arabidopsis*, providing important clues to the physiological functions of plant peroxisomal ABC transporters and their underlying biochemical bases (Zolman *et al.*, 2001; Footitt *et al.*, 2002; Hayashi *et al.*, 2002; Hooks *et al.*, 2007). The *cts-1* mutant was identified in a screen for genes which control germination and is also impaired in seedling establishment (Russell et al., 1998; Footitt *et al.*, 2002). Whilst the establishment phenotype reflects the inability to mobilize storage lipid and can be rescued with exogenous sucrose (Zolman *et al.*, 2001; Footitt *et al.*, 2002; Hayashi *et al.*, 2002), fatty acid mobilization is not required for germination (Pinfield-Wells *et al.*, 2005; Footitt *et al.*, 2006; Kelly *et al.*, 2011). The germination defect of *cts* mutants was recently shown to be associated with accumulation of the JA precursor 12-oxophytodienoic acid (OPDA) which triggers up-regulation of the ABSCISIC ACID-INSENSITIVE 5 (ABI5) transcription factor, leading to inhibition of seed coat rupture (Kanai *et al.*, 2010; Dave *et al.*, 2011). CTS is thought to mediate OPDA transport into the peroxisome, since null mutants are JA deficient, but a second, minor import pathway also exists, as *cts* mutants are not male sterile (Theodoulou *et al.*, 2005). It is possible that OPDA could enter the peroxisome by passive diffusion and be subject to vectorial acylation involving peroxisomal OPDA:CoA ligase (Kienow *et al.*, 2008), though an as yet unidentified transporter cannot be ruled out.

Alleles of *cts* have been isolated in screens for resistance to the natural auxin, indole-3-butyric acid (IBA) and the artificial pro-auxin, 2,4-dichlorophenoxybutyric acid (2,4-DB) (*pxa1-1*, Zolman *et al.*, 2001; *ped3* series, Hayashi et al., 1998, 2002). These compounds are metabolized by one round of β-oxidation to generate IAA and 2,4-dichlorophenoxyacetic acid (2,4-D), respectively, which inhibit root and hypocotyl growth (Hayashi *et al.*, 1998; Zolman *et al.*, 2001; Rashotte *et al.*, 2003). This implies that CTS can transport these aromatic compounds or their CoA esters, although this has not been tested experimentally. Finally, a *cts* allele (*acn2*) was shown to be resistant to fluoroacetate, implying a role in acetate metabolism (Hooks *et al.*, 2007). Thus, it appears that plants have apparently evolved a broad specificity peroxisomal ABC transporter to mediate the import of the wide range of substrates that they need to process by β-oxidation (Baker *et al.*, 2006). This is in contrast to mammalian and yeast peroxisomal ABC transporters which exhibit restricted substrate selectivities as judged by cross-kingdom complementation experiments and characterization in yeast and *in vivo* (van Roermund et al., 2008, 2011, 2014; Zhang *et al.*, 2011).

With the exception of a study implicating a *CTS* homologue in the control of seed size in tomato (Orsi and Tanksley, 2009), ABCD proteins have not been investigated in plant species other than *Arabidopsis*, and it remains to be determined to what extent the functions identified thus far are shared with other taxa and whether their relative importance differs. *Arabidopsis* and other oilseeds utilize oil stored primarily in cotyledons as the main source of energy during seedling establishment. In contrast, in cereals such as barley, starch stored in the endosperm fulfils this role, although the embryo, scutellum, and aleurone contain significant amounts of stored lipid (Jones and Jacobsen, 1991). Tissue-specific transcriptome analysis has shown that genes of the β-oxidation pathway are induced in both endosperm/aleurone and embryo from 24h after imbibition of barley grains (Sreenivasulu *et al.*, 2008), and enzymes of β-oxidation are present in both tissues. Gibberellic acid (GA) stimulates the breakdown of oil reserves and their conversion to sugar by gluconeogenesis in cereal aleurone (Doig et al, 1975; Newman and Briggs, 1976; Fernandez and Staehelin, 1987), although sensitivity to GA is cultivar dependent (Eastmond and Jones, 2005). It has been proposed that mobilization of stored triacylglycerol (TAG) from aleurone tissue provides energy and carbon skeletons for the synthesis of α-amylase that is needed to mobilize starch. An alternative hypothesis is that sucrose provided by gluconeogenesis in the aleurone could be transferred to the growing embryo via the scutellum, to support embryo growth (Eastmond and Jones, 2005). Although the embryo and scutellum are rich in oil, these tissues lack glyoxylate cycle enzyme activities (Holtman *et al.*, 1994), and thus may respire fatty acids, rather than use them for gluconeogenesis (Eastmond and Jones, 2005). In conclusion, β-oxidation might play key roles in barley germination, an important trait in agriculture and malting.

In this study, the roles of peroxisomal ABC transporters in a model cereal, barley (*Hordeum vulgare* L.), were investigated and experiments were carried out to test whether they perform similar functions to those in oilseeds such as *Arabidopsis*. The isolation of two barley CTS homologues, *HvABCD1* and *HvABCD2*, is reported and their collective roles are analysed using RNA interference (RNAi). In parallel, the individual functions of *HvABCD1* and *HvABCD2* were investigated by testing their ability to complement the *Arabidopsis cts-1* mutant and the yeast *pxa1/pxa2Δ* mutant which lacks the homologous transporter. Together, these approaches enabled the physiological roles of barley peroxisomal ABC transporters to be probed and the contributions of the two different genes to different known biochemical functions to be assessed. It is concluded that the general capabilities of peroxisomal ABC transporters of *Arabidopsis* and barley are similar but that the two paralogues in barley may play distinct roles.

## Materials and methods

### Plant material and growth conditions

Barley, *Hordeum vulgare* L. var. Golden Promise, was grown under controlled conditions of 15 °C/12 °C (day/night) and a 16h photoperiod [80% relative humidity, 500 μmol m^–2^ s^–1^ metal halide lamps (HQI) supplemented with tungsten bulbs]. Seeds were sown in 5 litre pots containing Levington’s C2 compost (http://www.everris.com/uk/Home/Ornamental-Horticulture/Products/Product.aspx/Professional-Growing-Media/Potting-and-Pot-Plant-Compost/_/_/MCP, last accessed 21 May 2014). Heads were harvested at maturity, dried for 7 d, and threshed by hand to prevent damage to the husk and embryo.

### Identification and cloning of *HvABCD1* and *HvABCD2*

Analysis of the rice genome and barley expressed sequence tag (EST) sequences revealed two *CTS* homologues in grasses. The barley genes were designated *HvABCD1* and *HvABCD2*, according to nomenclature conventions prescribed in Verrier *et al.* (2008). *HvABCD1* was amplified from barley cDNA using primer pair CTS-a startF/Hv14973R2 (Supplementary Table S1 at *JXB* online) and blunt-end cloned into pBluescript, to give pCTS1/BS. Plasmids for the expression of *HvABCD1* and *HvABCD2* under the control of the native *CTS* promoter were constructed as follows: first, the promoter region of *AtCTS* was amplified from pG0229-T ProGFP (Footitt *et al.*, 2007a) with primer pair AtCTSp5′Nco/AtCTSp3′Kpn, excised with *Nco*I/*Kpn*I, and cloned in the corresponding sites of pENTR11 (Invitrogen), to give pE-AtCTSp. The open reading frame (ORF) of *HvACBD1* was amplified from pCTS1/BS using primer pair HvCTSa Kpn5′/HvCTSa Not3′ and cloned into the *Kpn*I/*Not*I sites of pE-AtCTSp. Following confirmation of the insert by sequencing, the entry clone was recombined with pKanGWFS7 (Karimi *et al.*, 2002) using Gateway technology (Invitrogen), to give pAtCTS:HvABCD1. *HvABCD2* was amplified in a nested PCR strategy. The first 670bp of *HvABCD2* cDNA was amplified from barley cDNA with primers CTSb Kpn5′/CTSb NotI-BglII R and cloned in the *Kpn*I/*Not*I sites of pE-AtCTSp to create pE-CTSb5′. The remaining cDNA was amplified from 24h imbibed barley embryo cDNA, using primer pair CTSb Bgl IIF/CTSb Not3′ and cloned into the *Bgl*II/*Not*I sites of pE-CTSb5′. The inserts were confirmed by sequencing and the two entry clones were recombined into pGWB7 (Nakagawa *et al.*, 2007) to give pAtCTS:HvABCD2.

### Genetic mapping

The cDNA sequences of *HvABCD1* and *HvABCD2* were BLASTed against the recently released sequence assembly of the barley cv. Morex genome (IBSC, 2012) identifying corresponding genomic sequence contigs (*HvABCD1*=morex_contig_47855, *HvABCD2*=morex_contig_368523). Both contigs have been integrated into a genetically ordered sequence context using POPSEQ (Mascher *et al.*, 2013) in two biparental segregating populations: the reference Morex×Barke recombinant inbred line (RIL) population and OWB doubled haploid population (IBSC 2012). Map positions are related to the positions of the barley iSelect markers as reported in Comadran et al (2012).

### Reverse transcription–PCR (RT–PCR)

**Fig. 1.**
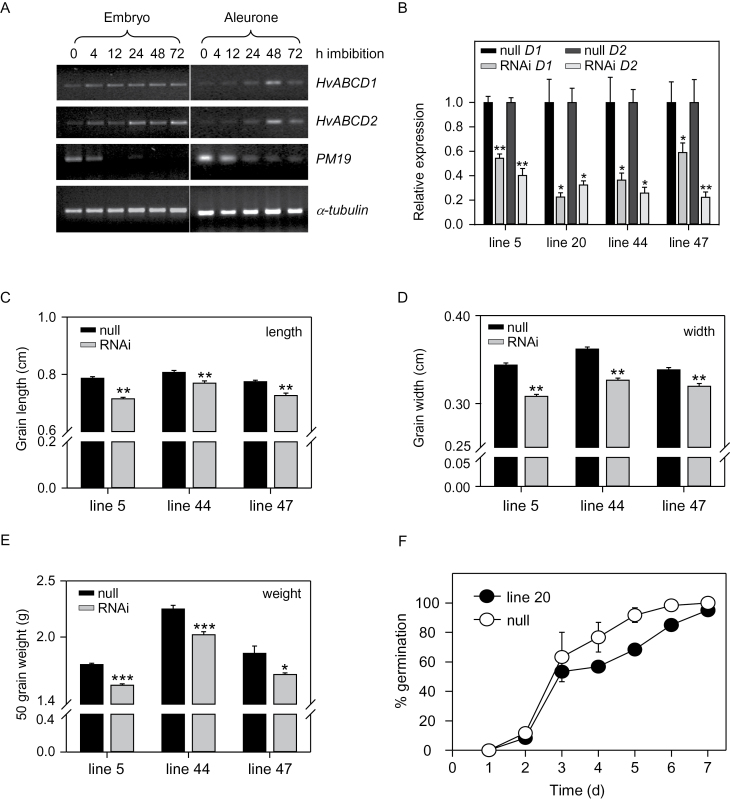
Seed phenotypes of *HvABCD*i lines. (A) RT–PCR analysis of *HvABCD1* and *HvABCD2* expression in embryo and endosperm during germination. (B) Quantitation of *HvABCD1* and *HvABCD2* transcripts in RNAi lines, using Q-PCR. Transcript abundance in each RNAi line is expressed relative to abundance in the respective null segregant. Values are means ±SE (*n*=3); *P<0.05, **P<0.01. (C–E) Grain length, width, and weight in RNAi lines, relative to the respective null segregants. Values are means ±SE (*n*=5, 50 seeds per replicate); *P<0.05, **P<0.01, ***P<0.001. (D) Germination of RNAi line 20 and the corresponding null segregant over 7 d. Values are means ±SE (*n*=3).

For the experiment presented in Fig. 1A, embryos and aleurone were isolated from barley grains and incubated in water for different periods (0, 4, 12, 24, 48, and 72h). RNA was extracted from embryos using a modified TRIZOL extraction protocol, and aleurone tissue RNA was extracted according to Singh *et al.* (2003). Poly(dT) cDNA was prepared from 2 μg of total RNA with SuperScript III reverse transcriptase (Invitrogen). Amplification was conducted using the primer pairs HvABCD1 F/R and HvABCD2 F/R; cycle conditions: 96 °C 2min; 30 cycles of (94 °C 30 s, 55 °C 30 s, 72 °C 30 s); 72 °C 7min. Constitutively expressed *α-tubulin* was used as a loading control (Nicot *et al.*, 2005; Jarošová and Kundu, 2010) and *PM19* was used as a developmental control (Ranford *et al.*, 2002). Amplifications were conducted using the primer pairs α TUB F/R [cycle conditions: 96 °C 2min; 25 cycles of (94 °C 30 s, 58 °C 30 s, 72 °C 30 s); 72 °C 7 min] and PM19 F/R [cycle conditions: 96 °C 2min; 30 cycles of (94 °C 30s, 55 °C 30 s, 72 °C 30 s) ×30; 72 °C 7 min], respectively.

### Generation of RNAi lines

A 497bp fragment of *HvABCD2* with high sequence identity to *HvABCD1* (Supplementary Fig. S1 at *JXB* online) was amplified from cDNA using primers HvABCDRNAiF and HvABCDRNAiR, and recombined sequentially into pGWC (kind gift of Dr Ranjan Swarup, University of Nottinhgam) and pBract207, via Gateway cloning (Invitrogen). Transgenic barley plants (var. Golden Promise) were generated by the BRACT transformation facility (John Innes Centre, Norwich, UK), using *Agrobacterium*-mediated transformation of immature embryos, according to the method of Harwood *et al.* (2009). Copy number and zygosity determination were carried out by IDna GENETICS (Norwich, UK).

### Analysis of RNAi lines

#### Quantitative real-time PCR (Q-PCR)

RNA was extracted from seedling leaf material (Zadoks code 15, which corresponds to five visible leaves), using a Nucleospin RNA plant mini-kit (Macherey-Nagel, Germany). Leaf material (70–100mg) was ground to a powder in liquid N_2_ and resuspended in 400 μl of RA1 extraction buffer, containing 1% (w/v) PVP-40. Following clarification by centrifugation, the supernatant was processed according to the manufacturer’s instructions. Poly(dT) cDNA was prepared from 1mg of total RNA with SuperScript III reverse transcriptase (Invitrogen), using primers as specified in Supplementary Table S1 at *JXB* online. Quantitative PCR was performed using SYBR-green Sensimix (Bioline), in 384-well optical reaction plates with a Roche LightCycler 480 apparatus (Roche/Applied Biosystems). PCR conditions and quantification were as specified by the manufacturer (Roche). The relative number of copies obtained for each transcript was normalized against *HvELF1* and *HvTubulin* (Nicot *et al.*, 2005; Jarošová and Kundu, 2010) or *AtCTL1* and *AtCTL3* (De Rybel *et al.*, 2010) transcript values for each sample as an internal reference.

#### Seed size measurement

Five samples of 50 grains from homozygous *HvABCD1/2* RNAi lines and their respective null segregants were weighed. Seeds were photographed using a camera attached to a light microscope. Length and width were determined from micrographs, following correction for magnification.

#### Germination assays

Fifty grains were placed in Petri dishes containing two layers of Whatman No. 2 filter paper and 4ml of water. The dishes were sealed with Micropore tape and incubated at 22 °C under continuous white light (150 μmol m^–2^ s^–1^). Germinated caryopses, defined by the emergence of coleorhizae beyond the seed coats, were scored every 24h over 7 d and removed from the dishes. Assays were performed in triplicate.

#### Root growth assays

Grains from homozygous *HvABCD1/2* RNAi lines and their respective null segregants were plated on 0.5× Murashige and Skoog (MS) medium with 0.5% (w/v) sucrose containing the indicated concentrations of 2,4-DB or IBA, or no supplements. Experiments were carried out in triplicate with four grains per replicate.

#### JA treatment

Seedlings of *HvABCD1/2* RNAi lines were grown in a growth chamber (20 °C day/15 °C night, 70% relative humidity, 16h light). Ten-day-old plants were sprayed with 2ml of methyl jasmonate (Sigma-Aldrich, Germany) at 2mg ml^–1^ in water. After 48h, the treatment was repeated and leaf material was sampled 72h after the first treatment. RNA extraction and Q-PCR were performed as described above.

### Scanning electron microscopy

Intact grains were imbibed in sterile distilled water (SDW) for 2–5 d, on moistened filter paper sealed in Petri dishes and placed in the dark at room temperature. Following imbibition, root and shoot were removed if present and the remaining tissue was mounted onto cryo electron microscope stubs using OCT compound (Agar Scientific UK) and plunge-frozen in pre-slushed liquid N_2_. They were then transferred under vacuum to the Alto 2500 (Gatan UK) cryo chamber stage which was pre-cooled to –180 °C. Here they were fractured using the cold blade mounted in the chamber and gentle etching was performed through sublimation by raising the temperature of the stage to –95 °C for 2min. The stage temperature was returned to –140 °C and the samples were sputter-coated with AuPd for 60 s to a thickness of ~10nm. Samples were transferred to the JEOL JSM 6700 scanning electron microscope (SEM) chamber and mounted on the stage cooled to –150 °C, for examination. This temperature was maintained during examination of the fractured surface, and images were recorded using the on-board system and software.

### Confocal microscopy

Grains were de-husked and de-embryonated using a sterile scalpel before sterilizing by immersion in 20% (v/v) sodium hypochlorite for 10min. The samples were thoroughly washed for 1min under a flowing stream of SDW. Each grain was placed in a 1.5ml Eppendorf tube and covered with ~1ml of 1 μM GA_3_, and left to imbibe at room temperature for 8–96h. At the required time points, grains were washed in SDW and dried on filter paper to remove excess water. Grains were individually mounted onto cryostat holders using Tissue-Tek, OCT compound (Agar Scientific UK) and were quickly plunged into liquid N_2_. When bubbling ceased, the frozen samples were transferred to the chamber of the Leica 1850 Cryostat (Leica Microsystems UK) and left for 30min to allow the temperature to equilibrate to the chamber temperature of –20 °C before sectioning. Sections were removed from the sample and discarded until the mid-region was reached ~1.6mm from the tip. The following 10 sections, each 16 μm thick, were collected on glass slides for staining. Sections were stained using Nile Red at 1 μl ml^–1^ for 1min, followed by washing with SDW and sealing with a cover slip. Sections were imaged using a Zeiss LSM 780 system, with laser 514nm selected and emission collected between 539nm and 753nm.

### *Arabidopsis* complementation

Wild-type (L*er*) and *cts-1* plants were transformed with pAtCTS:HvABCD1 and pAtCTS:HvABCD2 by floral dip. Lines expressing *HvABCD2* in the *cts-1* background were obtained by selecting *cts-1* transformants which germinated on 0.5× MS medium and confirming genotype by selection and PCR analysis. As *HvABCD1* did not complement the germination phenotype of *cts-1* (see the Results), transgenic lines were obtained in the L*er* background, crossed to *cts-1* and a homozygous line identified by PCR genotyping. It was not possible to produce multiple independent homozygous lines for *HvABCD1.* Assays for germination, establishment, and hypocotyl growth were carried out as described in Dietrich *et al.* (2009). Seed size was determined as described above for barley (30 seeds per line). The effect of OPDA on root growth was determined as described in Zhang and Turner (2008).

### Yeast complementation

*HvABCD1* was amplified with primer pair FT206/FT207, restricted with *Kpn*I/*Pst*I, and cloned into the corresponding sites of pEL30 and pIJL30. The inserts were sequenced to confirm the absence of PCR-generated mutations. Yeast transformation and oleate growth tests were carried out as described in Nyathi *et al.* (2010). β-Oxidation measurements were carried out as described in van Roermund *et al.* (1995); in brief, 1-^14^C-labelled fatty acids were supplied to intact cells followed by quantification of ^14^CO_2_ and ^14^C-labelled β-oxidation products by liquid scintillation counting.

## Results

### Barley has two *CTS* homologues which are expressed in embryo and endosperm

Comparative analysis of sequenced plant genomes has revealed that cereals contain two *CTS* homologues, consistent with a gene duplication occurring after divergence of the Gramineae family (Nyathi *et al.*, 2012). Full-length cDNAs corresponding to the barley *CTS* homologues were isolated by RT–PCR and designated *HvABCD1* and *HvABCD2*, according to the naming convention outlined in Verrier *et al.* (2008). *HvABCD1* and *HvABCD2* were located on the barley genome by BLASTing the cDNA sequence against a sequence assembly of the Morex genome that had been genetically ordered using the POPSEQ approach (Mascher *et al.*, 2013). *HvABCD1* was located at 148.4 cM on chromosome 3H on the Morex×Barke map. *HvABCD2* was located at 8.9 cM on chromosome 1H on the reference Morex×Barke RIL population map and 11.2 cM on 1H on the Oregon Wolfe Barley doubled haploid map (International Barley Sequencing Consortium, 2012). The orthologous genes in rice mapped to syntenic positions on the corresponding chromosomes.

Examination of *HvABCD1* and *HvABCD2* expression profiles using the Bio-Array Resource barley eFP browser (Patel *et al.*, 2012) revealed that both genes were widely expressed in different tissues, with *HvABCD2* transcripts being generally more abundant (Supplementary Fig. S2 at *JXB* online). The expression of *HvABCD1* and *HvABCD2* was then investigated in more detail in germinating barley grains over 3 d imbibition, using RT–PCR. *PM19*, which encodes a putative plasma membrane transporter, was used as a developmental control. Consistent with previous reports (Ranford *et al.*, 2002), transcripts were present in unimbibed seeds but declined upon germination (Fig. 1A). This confirmed the developmental status of the grains, since dormant embryos have been shown to retain high levels of *PM19* message for up to 72h of imbibition (Ranford *et al.*, 2002). *HvABCD1* transcripts were barely detectable in dry grains but were present in embryos at a similar level from 4h to 72h imbibition. *HvABCD2* expression in embryos increased steadily over this period (Fig. 1A). Both genes were expressed transiently in aleurone tissue at 48h imbibition, corresponding to a time point around germination. This suggests that β-oxidation could be important for embryo and aleurone function during germination and seedling establishment in barley.

### Suppression of *HvABCD1* and *HvABCD2* affects grain size but not germination

In order to understand the function of CTS homologues in barley growth and development, expression of both *HvABCD* genes was reduced simultaneously using an RNAi approach. Barley (cv. Golden Promise) was transformed with a construct designed to suppress both *HvABCD* transcripts (Supplementary Fig. S1 at *JXB* online), driven by the constitutive *Ubi1* promoter (Harwood *et al.*, 2009). Several *HvABCD1/2* RNAi lines (hereafter referred to as *HvABCD1/2*i lines) were obtained and, for each one, a null segregant line was also selected for use as a control. Expression levels were analysed by Q-PCR analysis of leaf material. Abundance of *HvABCD1* and *HvABCD2* transcripts varied from 22% to 59% and 22% to 40% of levels measured in corresponding null segregant lines, respectively (Fig. 1B).

Since natural variation in seed size has been associated with *ABCD* transporter genes in tomato and *Arabidopsis*, and *cts* alleles have small seeds (Russell, 1998; Alonso-Blanco *et al.*, 1999; Orsi and Tanksley, 2009), it was investigated whether suppression of *HvABCD1* and *HvABCD2* affected grain size in barley. Grains of three different RNAi lines exhibited significantly reduced length, width, and weight, compared with null segregants (Fig. 1C–E). The germination of seeds from several *HvABCD1/2*i lines and their respective nulls was also measured over 7 d. Although the percentage germination of null lines at 7 d was somewhat variable, in none of the RNAi lines tested was germination markedly different from that of the respective null segregant (Fig. 1F; Supplementary Fig. S3 at *JXB* online). This is in agreement with results from tomato, where ABCD function does not appear to affect the rate of germination (Orsi and Tanksley, 2009), but in contrast to *Arabidopsis*, in which *cts* mutants are arrested late in phase II of germination (Russell *et al.*, 2000; Footitt *et al.*, 2002, 2006; Carrera *et al.*, 2007).

### Suppression of *HvABCD1* and *HvABCD2* does not markedly affect seedling establishment and lipid mobilization

**Fig. 2.**
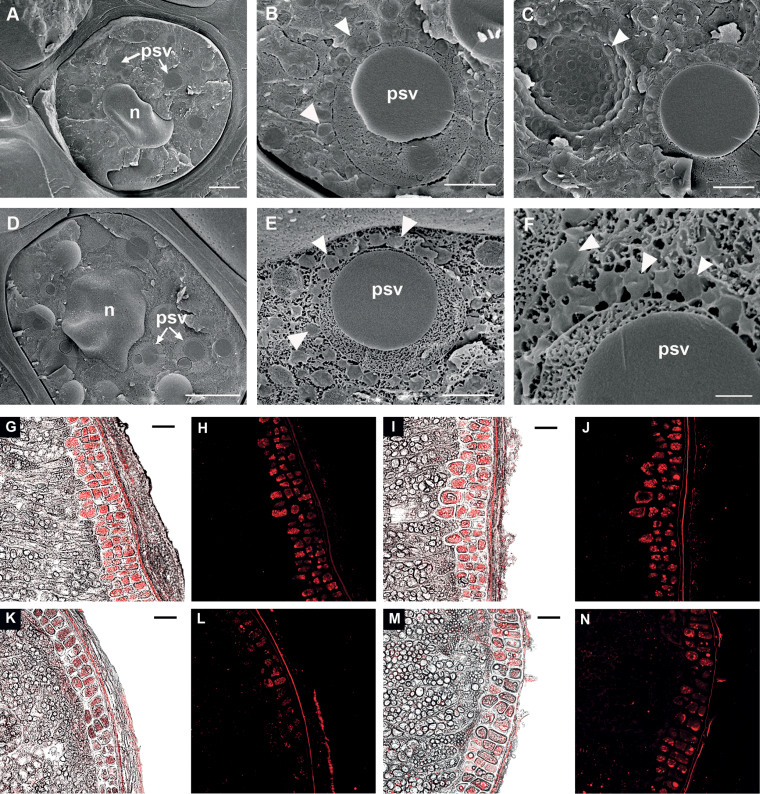
Oil body morphology in barley grains. (A–F) SEM cryo-fractured images of wild-type (var. ‘Golden Promise’) barley aleurone cells. (A–C) Two day imbibed grains; (D, E), 5 d imbibed grains. (A, D) Aleurone cells, showing nucleus (n) and numerous protein storage vacuoles (psv). (B, E) PSV, surrounded by oil bodies (OBs), indicated by arrowheads. (C) PSV fractured across, surrounded by OBs; the arrowhead indicates the extracellular face (‘E-face’) where PSV and OBs have been removed during fracture. (F) Close-up of (E), showing connections between OBs and PSV. OBs are indicated by arrowheads. (G–N) Confocal images of unfixed barely grain sections showing Nile Red staining of OBs in aleurone cells. De-embryonated grains were imbibed for 24h (G–J) or 96h (K–N) in the presence of 1 μM GA. (G, H, K, L) *HvABCD1/2* RNAi line 5; (I, J, M, N) null segregant. In G, I, K and M, the confocal image is overlaid on the bright field image. Scale bars: A=5 μm, B, C, E=1 μm; D=4 μm; F=400nm; G–N=50 μm.

In *Arabidopsis*, peroxisomal β-oxidation plays a critical role in mobilization of storage reserves during seedling establishment (Theodoulou and Eastmond, 2012). However, seedling establishment was not visibly affected in *HvABCD1/2*i lines, which is perhaps unsurprising, given that barley contains abundant reserves of starch in the endosperm. Nevertheless, since mobilization of TAG stored in the cereal aleurone has been suggested to facilitate the exploitation of endosperm starch and/or to support embryo growth (Eastmond and Jones, 2005), oil body morphology was examined over 5 d imbibition. After 2 d imbibition, scanning electron microscopy revealed that aleurone cells of wild-type cells contain numerous protein storage vacuoles (PSVs), surrounded by oil bodies (Fig. 2A–C). The oil body membrane is continuous with the PSV membrane, as previously reported (Fernandez and Staehelin, 1987); this is particularly evident where freeze-fracture has generated images showing extracellular (E-)faces with ‘scars’ where oil bodies have been removed along with PSVs (Fig. 2C). After 5 d, numerous oil bodies were still visible, though they appeared less spherical (Fig. 2D–F). It was also possible to visualize aleurone oil bodies by confocal microscopy using Nile Red staining of TAG in unfixed cryo-sections of de-embyronated grains treated with GA (Fig 2G–N). After 5 d, the abundance of oil bodies had declined (Fig. 2K–N), in agreement with reports that GA stimulates lipid mobilization in this tissue (Fernandez and Staehelin, 1987; Eastmond and Jones, 2005). Since it was not possible to derive quantitative data from these images, fatty acids were quantified in de-embryonated, GA-treated barley grains, but were not found to be significantly different in RNAi and null segregant lines (data not shown).

### HvABCD proteins play roles in auxin and jasmonate metabolism

**Fig. 3.**
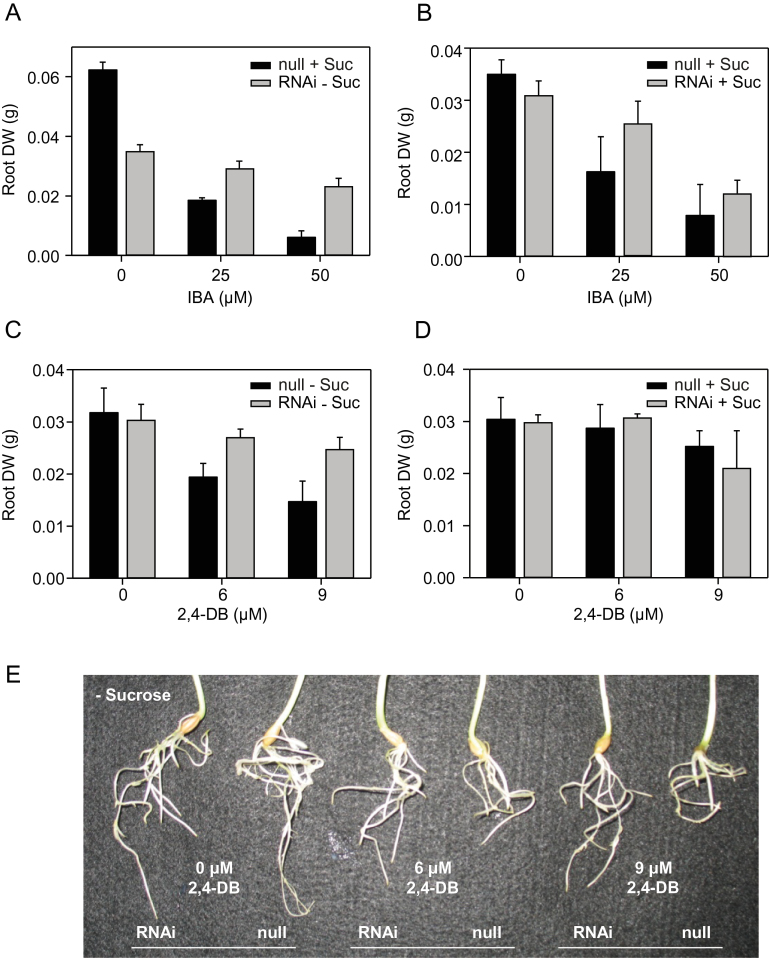
Root responses of *HvABCD*i lines to auxin precursors. (A) Dry weight of RNAi line 5 roots grown in different concentrations of indole-3-butyric acid (IBA). (B) As A, in the presence of 0.5% sucrose. (C) Dry weight of RNAi line 20 roots grown in different concentrations of 2,4-dichlorophenoxybutyric acid (2,4-DB). (D) As C, in the presence of 0.5% sucrose. Values are means ±SE (*n*=4). (E) Images of roots of RNAi line 5 and the corresponding null segregant, grown in medium lacking sucrose, containing different concentrations of 2,4-DB. Data are representative of experiments with different lines. (This figure is available in colour at *JXB* online.)

It was next investigated whether barley ABCD transporters are required for β-oxidation of auxins, as has been shown for CTS (Zolman *et al.*, 2001; Hayashi *et al.*, 2002). Both IBA and 2,4-DB treatment inhibited root growth, as judged by the reduced root dry weight of null segregant lines (Fig. 3). The response was dose dependent, although higher levels of the hormones were required to inhibit growth, compared with *Arabidopsis* (Dietrich *et al.*, 2009). In the absence of sucrose, *HvABCD1/2*i lines exhibited resistance to IBA and 2,4-DB (Fig. 3A, C, E), suggesting that one or both of the barley transporters mediate import of these compounds into the peroxisome for β-oxidation. Interestingly, the inhibitory effect of IBA and 2,4-DB on barley root growth was reduced in the presence of sucrose (Fig. 3B, D), unlike in *Arabidopsis* where sucrose potentiates the effect of these compounds (Dietrich *et al.*, 2009). Because exogenous sucrose inhibits lipid mobilization in *Arabidopsis* seedlings (Martin *et al.*, 2002; Fulda *et al.*, 2004), the effect of sucrose on pro-auxin toxicity has been interpreted to imply a direct competition between fatty acids and IBA or 2,4-DB for ABCD-mediated transport (Dietrich *et al.*, 2009). The lack of such an effect in barley roots may indicate that the rate of fatty acid β-oxidation is not very high in this system or, alternatively, that sucrose promotes root growth and that this effect outweighs the inhibitory effect of auxins.

**Fig. 4.**
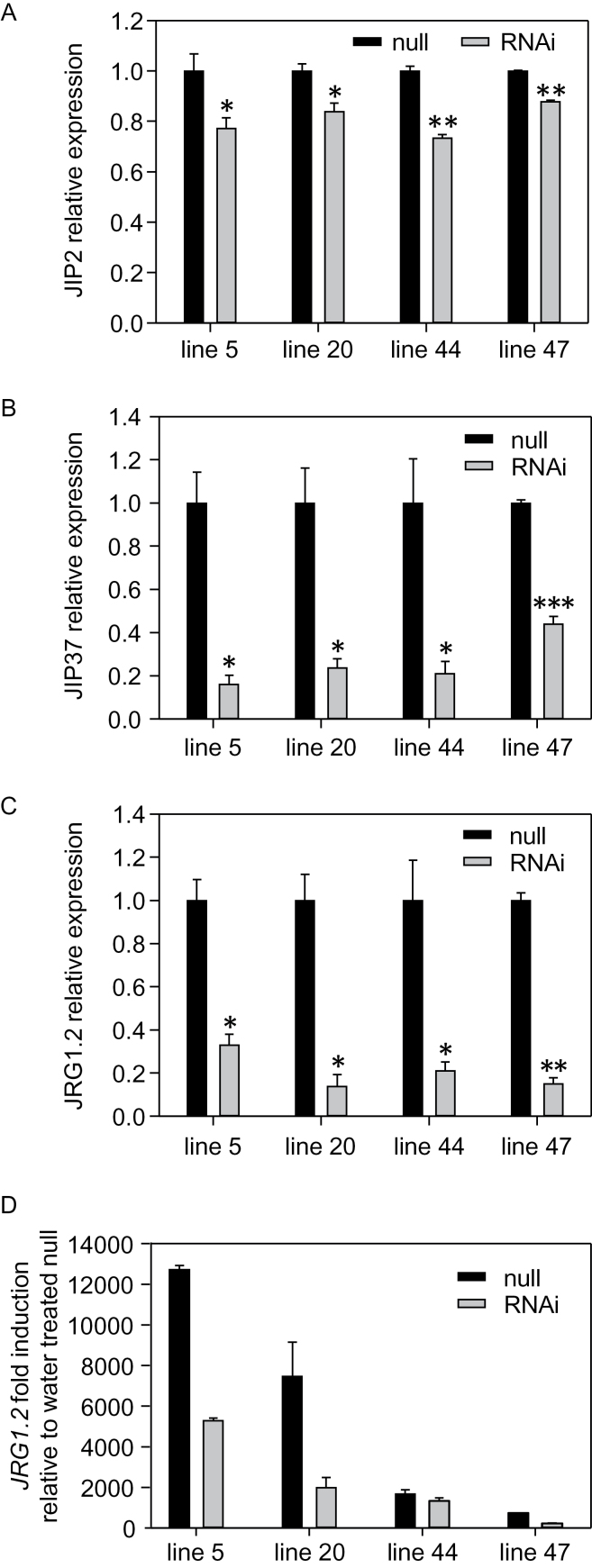
Expression of JA-responsive genes in *HvABCD*i lines. (A–C) Quantitation of *JIP2*, *JIP37*, and *JRG1.2* transcripts in RNAi lines, using Q-PCR. Transcript abundances are expressed relative to the respective null segregant lines. Values are means ±SE (*n*=3); data are representative of two independent experiments. *P<0.05, **P<0.01, ***P<0.001. (D) Effect of exogenously applied methyl jasmonate (2mg ml^–1^) on expression of *JRG1.2* in RNAi lines and the respective null segregants. Bars show transcript abundance relative to water-treated nulls. Values are means ±SD (*n*=3).

ABCD transporters have also been implicated in import of OPDA for JA biosynthesis in *Arabidopsis*: *cts* mutants exhibit reduced levels of JA and lower expression of the JA-responsive gene, *VSP2* (Theodoulou *et al.*, 2005). Therefore, the expression of three barley genes which respond to endogenous JA production, *JIP2*, *JIP37*, and *JRG1.2* (Kramell *et al.*, 2000; Walia *et al.*, 2007), was tested. Although the level of expression varied for different genes tested, in each case, expression was reduced in leaves of *HvABCD1/2*i lines, compared with null segregants (Fig. 4A–C). This result is consistent with a role for either or both of these genes in JA biosynthesis. Spraying with methyl jasmonate confirmed that *JRG1.2* expression was induced by exogenous hormone treatment in both controls and RNAi lines, though to a lesser extent in the latter (Fig. 4D).

### *HvABCD1* and *HvABCD2* differ in their ability to complement different phenotypes of *Arabidopsis cts-1*

Whilst analysis of the barley *HvABCD1/2*i lines permitted assignment of several different physiological roles to peroxisomal β-oxidation (Figs 1–4), this approach does not provide information about the relative contributions of *HvABCD1* and *HvABCD2* to these processes. In order to probe their individual functions, *HvABCD1* and *HvABCD2* were expressed in the *Arabidopsis cts-1* null mutant under the control of the native *CTS* promoter, as has been carried out previously for site-directed *cts* mutants (Dietrich *et al.*, 2009). Two homozygous lines (D2.1 and D2.2) expressing *HvABCD2* were obtained following transformation of the *cts-1* mutant. However, it was not possible to recover lines for *HvABCD1* by this method, as seeds were unable to germinate. Therefore, a line was established in the wild-type background (L*er*) and crossed to *cts-1*. A homozygous *cts-1* line expressing *HvABCD1* (D1.1) was recovered by mechanically disrupting the seed coat and culturing on sucrose-containing medium. All lines produced transcripts, as measured by Q-PCR analysis of seedlings; however, expression of *HvABCD1* was low relative to the expression level of endogenous *CTS* (Supplementary Fig. S4 at *JXB* online).

**Fig. 5.**
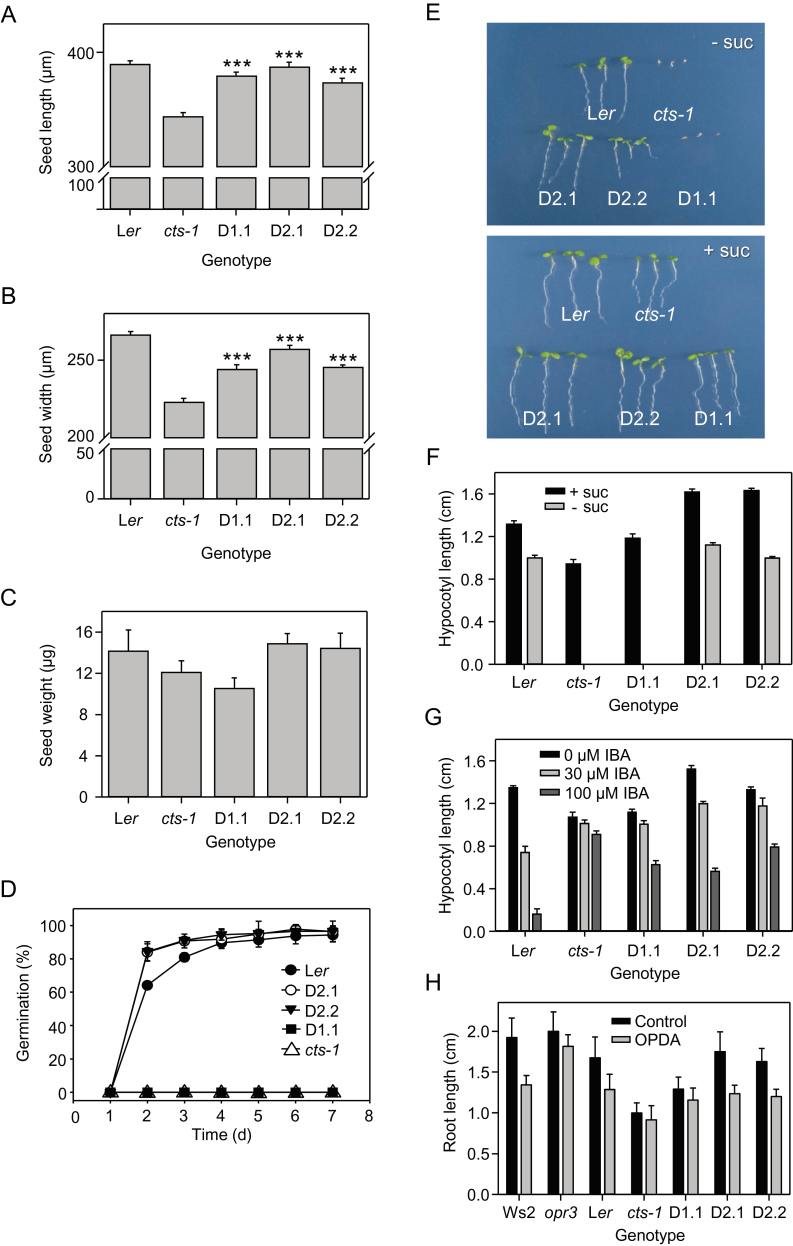
Complementation of *cts-1* by *HvABCD1* and *HvABCD2*. (A, B) Length and width of seeds of *cts-1*, complemented lines, and the wild type (L*er*). Values are means ±SE (*n*=3; 30 seeds per replicate). *t*-test indicates significant difference of transgenic lines from *cts-1* (***P<0.001). (C) Seed weight. Values are means ±SE (*n*=5; 80 seeds per replicate). (D) Germination over 7 d on water agarose. Values are means ±SE (*n*=3; 50 seeds per replicate). (E) Seedling establishment on 0.5× MS with or without 0.5% sucrose. In the upper panel, seeds of *cts-1* and *cts-1 AtCTS:HvABCD1.1* were induced to germinate by mechanically rupturing the seed coat and plating on sucrose medium for 2 d before transfer to medium lacking sucrose (–suc). Seedlings were rearranged on a fresh plate for photography. (F) Elongation of hypocotyls in the dark on 0.5× MS with or without 0.5% sucrose. (G) Elongation of hypocotyls in the dark on 0.5× MS containing 0.5% sucrose and different concentrations of IBA. Values are means ±SE (*n*=3; 20 hypocotyls per replicate) in F and G. (H) Effect of oxophytodienoic acid (OPDA) on root growth. Medium is 0.5× MS containing 0.5% sucrose; values are means ±SE (*n*=25). The *opr3* mutant lacks oxophytodienoate reductase and is unable to convert OPDA to JA. (This figure is available in colour at *JXB* online.)

Seed size was first measured in different genotypes. *cts-1* seeds were smaller than those of L*er* plants produced under identical conditions, being on average 88% of the length and 84% of the width of the wild type (Fig. 5A, B). This was accompanied by a reduction in average seed weight to 85% of the wild type, although this difference was not statistically significantly different (Fig. 5C). In *cts-1* lines expressing *HvABCD2*, seed length and weight were restored to wild-type values, and seed width was significantly increased relative to *cts-1* but still significantly different from L*er*. Complementation was less complete in the line expressing *HvABCD1* (Fig. 5A–C). Germination kinetics of lines D2.1 and D2.2 were indistinguishable from those of the wild type, whereas line D1.1 did not germinate, in agreement with the inability to recover transgenic lines via direct transformation of the *cts-1* mutant (Fig. 5D). Thus *HvABCD2* but not *HvABCD1* is able to complement *cts-1* germination. Since CTS performs distinct biochemical functions in germination and seedling establishment, the ability of transgenic lines to establish in the absence of sucrose was then tested. Sterilized seeds were plated on 0.5× MS medium, chilled for 2 d, and transferred to a growth chamber. Lines D2.1 and D2.2 produced green cotyledons after 5 d in constant light and were able to extend hypocotyls in the dark (Fig. 5E, F), although line D2.2 did not grow as well as the wild type (Fig. 5E). Seeds of line D1.1 were induced to germinate as described above and then transferred to medium lacking sucrose, but were unable to mobilize their storage lipid, as indicated by their inability to expand cotyledons and hypocotyls. However, development appeared normal in the presence of sucrose, indicating that there were no obvious deleterious effects of the transgenes (Fig. 5E, F).

Responses of the transgenic lines to hormones were also investigated. Seeds were induced to germinate and the effect of IBA on hypocotyl length was tested in sucrose-containing medium. As hypocotyls are less sensitive to IBA than roots (Rashotte *et al.*, 2003), this assay is less influenced by transporter expression levels (discussed in Dietrich *et al.*, 2009). Under these conditions, L*er* exhibited a dose-dependent inhibition of hypocotyl elongation, whereas *cts-1* was largely resistant up to 100 μM IBA (Fig. 5G). Partial sensitivity to 100 μM IBA was restored in *cts-1* lines expressing either *HvABCD1* or *HvABCD2*, indicating that both transporters can contribute to auxin metabolism, as suggested by the barley RNAi lines. The effect of the jasmonate precursor OPDA on root elongation was also tested (following rescue of germination for *cts-1* and *cts-1 pAtCTS:HvABCD1* seeds, as described above). OPDA inhibited elongation of wild-type (L*er*) roots, but the *opr3* mutant was resistant since it lacks 12-oxophytodienoic acid reductase and is unable to convert OPDA to JA (Stintzi and Browse, 2000; Zhang and Turner, 2008; Fig. 5H). *cts-1* roots were shorter than those of L*er* under control conditions, but were also resistant to OPDA, consistent with the fact that this mutant has been shown to be impaired in JA production (Theodoulou *et al.*, 2005). OPDA sensitivity was restored in roots of *cts-1* lines expressing *HvABCD2* but not in the line expressing *HvABCD1*.

### *HvABCD1* partially complements the yeast *pxa1/pxa2Δ* mutant for fatty acid β-oxidation

**Fig. 6.**
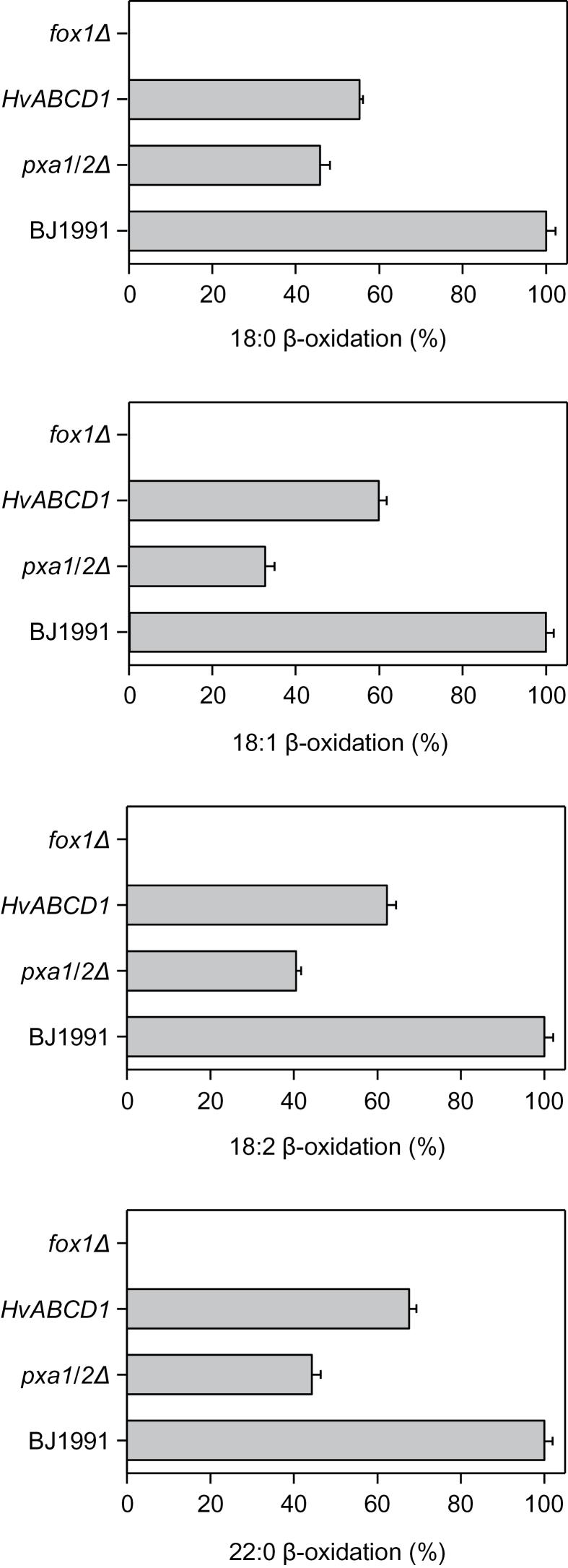
Complementation of *pxa1/pxa2Δ* by *HvABCD1*. β-oxidation of different fatty acids by intact yeast cells. Cells grown in oleate medium were incubated with 1-^14^C-labelled fatty acids, followed by β-oxidation activity measurements. Strains are: wild type (BJ1991), *pxa1/pxa2Δ* mutant cells transformed with vector lacking the insert, or *HvABCD1*. *fox1Δ* is defective in acyl-CoA oxidase (the first committed step of β-oxidation) and serves as a negative control. Results are presented as a percentage relative to the rate of oxidation by wild-type cells for each fatty acid and are means ±SD of three independent experiments.

Since *HvABCD1* did not complement the seedling establishment phenotype of *cts-1*, a role for this transporter in fatty acid metabolism was investigated further by expression in a *Saccharomyces cerevisiae* mutant which lacks Pxa1p/Pxa2p, the yeast heterodimeric transporter which is homologous to CTS. *pxa1/pxa2Δ* is defective in growth on oleate (18:1) as a sole carbon source, but expression of *HvABCD1* under the control of the yeast catalase promoter restored growth to near wild-type levels (data not shown). *pxa1/pxa2Δ* cells transformed with *HvACBD1* exhibited increased β-oxidation of several different long chain fatty acids compared with cells transformed with vector lacking an insert, indicating that the barley transporter can accept saturated and unsaturated long chain fatty acyl-CoAs in this heterologous system (Fig. 6). Expression of *HvABCD2* was also attempted using a similar strategy, but no complementation of the *pxa1/2Δ* mutant was observed (data not shown).

## Discussion

Using two complementary approaches, evidence has been provided that barley and *Arabidopsis* peroxisomal ABC transporters share several functions. When interpreting phenotypes of transgenic plants, it is important to consider the level of suppression in barley RNAi lines and the level of *HvABCD* expression in *cts-1* complemented lines (Dietrich *et al.*, 2009). *Arabidopsis* plants heterozygous for *cts* germinate and are able to establish in the absence of sucrose, but are resistant to 2,4-DB and IBA (Hayashi *et al.*, 1998; Zolman *et al.*, 2001). Thus the auxin phenotypes are more sensitive to loss of function than germination and lipid mobilization in *Arabidopsis*. Therefore, a minor reduction in ABCD function in an RNAi line could lead to auxin resistance but might not impact markedly on other phenotypes. By this reasoning, a substantial knockdown of ABCD transporter expression is likely to be required to detect effects on lipid metabolism and perhaps also germination. However, the physiological thresholds which underpin the visible phenotypes may vary between barley and *Arabidopsis*, and the influence of the expression level on phenotypes of complemented plants is to some extent dependent on the substrate specificity of the heterologous transporters. Unfortunately, an antiserum raised to the relatively well-conserved C-terminus of *Arabidopsis* CTS, which recognizes both NBDs (Footitt *et al.*, 2002; De Marcos Lousa *et al.*, 2009), did not recognize barley CTS (data not shown); thus it was not possible to determine the level of HvABCD1 or HvABCD2 protein expression in transgenic lines. With these caveats in mind, several conclusions can be reached.

### ABCD transporters play a role in seed size determination in diverse species

Both barley RNAi lines and the complemented *Arabidopsis cts-1* lines support a role for ABCD transporters in control of seed size (Figs 1C–E, 5 A–C). This is in agreement with a study identifying a tomato orthologue as the cause of *Seed weight 4.1* (*Sw4.1*), a major quantitative trait locus (QTL) controlling seed size in the genus *Solanum* (Orsi and Tanksley, 2009). Although studies with *Arabidopsis* indicate that plant peroxisomal ABCD transporters have multiple substrates (Linka and Theodoulou, 2013), their precise biochemical role in seed size determination is unknown. However, reduced seed size is also observed in *Arabidopsis kat2* mutants which lack 3-ketoacyl CoA thiolase (Footitt *et al.*, 2007b), implying a general role for β-oxidation in this trait. Embryo development appears to be dependent on a functional β-oxidation pathway, as evidenced by increased ovule abortion in *cts* and *kat2* mutants (Footitt *et al.*, 2007a, b) and embryo lethality of *acox3 acox4* double mutants, which lack short chain acyl-CoA oxidase activity (Rylott *et al.*, 2003), although the latter phenotype is accession dependent (Khan *et al.*, 2012). In agreement with this, reciprocal crosses suggest that *Sw4.1* controls seed weight through zygotic effects and exerts its largest effect during early seed development (Orsi and Tanksley, 2009). Deposition of starch and lipids into endosperm cells is maximal at this stage, perhaps suggesting that β-oxidation might also be important for seed filling, either via provision of energy and carbon skeletons for endosperm reserve synthesis or possibly by hormonal control of this process. Seed size is a key agronomic trait and has increased during domestication in crops, as a result of selection for yield and harvest efficiency (Harlan *et al.*, 1973). Interestingly, in this context, attempts to engineer increased seed oil content by overexpression of oil biosynthetic genes in Brassicas has led to seeds of increased weight and/or size (Zou *et al.*, 1997; Jako *et al.*, 2001; Vigeolas *et al.*, 2007).

### *HvABCD2* contributes to OPDA metabolism

Transcript abundances of JA-inducible genes were reduced in leaves of barley RNAi lines, consistent with a role for *HvABCD* genes in conversion of OPDA to JA (Fig. 4). Whilst *HvABCD1* did not complement *cts-1* for germination, expression of *HvABCD2* restored the germination rate to wild-type levels (Fig. 5D), which is supportive of the proposal that HvABCD2 mediates OPDA transport into the peroxisome. In agreement with this, roots of *cts-1* lines expressing *HvABCD2* but not *HvABCD1* exhibited sensitivity to exogenously applied OPDA similar to that of wild-type controls (Fig. 5H), strongly supporting the notion that HvABCD2 is required for the conversion of OPDA to JA.

Although accumulation of OPDA is associated with inhibition of germination in *Arabidopsis* (Dave *et al.*, 2011), the influence of different jasmonates on barley germination has not been studied in detail. The effects of jasmonates on seed germination appear to be species and even ecotype specific (Linkies and Leubner-Metzger, 2012); however, a recent transcriptome study demonstrated that jasmonate biosynthetic and putative receptor genes are up-regulated in coleorhiza tissue of imbibed after-ripened seeds, relative to dormant seeds, pointing to the importance of this class of compounds in barley germination (Barrero *et al.*, 2009). Suppression of *HvABCD1* and *HvABCD2* had little or no effect on germination (Fig. 1F; Supplementary Fig. S3 at *JXB* online), perhaps suggesting that ABCD transport activity is not essential for this function in barley, as has also been proposed for tomato (Orsi and Tanksley, 2009). However, the level of *HvABCD* knockdown may have been insufficient to impact on germination in the present experiments, and it is also possible that, as in *Arabidopsis*, an additional peroxisomal import pathway for OPDA operates in barley seeds (Theodoulou *et al.*, 2005).

### *HvABCD1* and *HvABCD2* are functional in fatty acid β-oxidation

Oil bodies have been shown to occupy >40% of the cell volume in barley aleurone, and embryo-derived GA stimulates oil breakdown during germination (Eastmond and Jones, 2005). The TAG composition is similar in *Arabidopsis* and barley, with the exception that *Arabidopsis* contains a high level (~25%) of the very long chain fatty acid, eicosanoic acid (20:1) (Hølmer *et al.*, 1973; De Man and Vervenne, 1988; Lemieux *et al.*, 1990). In barley, genes involved in lipid catabolism and starch mobilization are expressed together with key transcripts of sucrose synthesis as early as 24h after imbibition before radical protrusion, suggesting that lipid reserve mobilization could supply sucrose before the seedling becomes photoautotrophic (Sreenivasulu *et al.*, 2008). Although oil bodies declined in abundance over 5 d of imbibition of intact grains and also in response to GA treatment of de-embryonated seeds (Fig. 2), increased oil body or fatty acid retention was not observed in *HvABCD1/2*i lines. It might have been predicted that suppressing *HvABCD1* and *HvABCD2* would result in retention of oil bodies, but it is possible that the RNAi lines are not sufficiently impaired in β-oxidation to give a detectable phenotype, consistent with the observation that *Arabidopsis* mutants in which lipid breakdown is reduced by 50% do not exhibit a marked growth phenotype (Kelly *et al.*, 2011). However, complementation of the *Arabidopsis cts-1* mutant for seedling establishment in the absence of sucrose indicates that HvABCD2 can transport a range of different fatty acid species (Fig. 5E, F). Although *HvABCD1* did not complement the seedling establishment phenotype of *cts-1*, it did partially restore fatty acid β-oxidation to the yeast *pxa1/pxa2Δ* mutant (Fig. 6). The lack of complementation *in planta* may reflect the low expression level of *HvABCD1* but could also result from differences between the two expression systems, such as the ability of the heterologous transporter to interact with the different endogenous peroxisomal acyl-CoA synthetases (van Roermund *et al.*, 2012; De Marcos Lousa *et al.*, 2013).

### HvABCD1 and HvABCD2 contribute to IBA metabolism

Barley RNAi lines (Fig. 3) and the complementation of *cts-1* (Fig. 5G) suggest a role for HvABCD proteins in IBA metabolism. IAA is synthesized by several different routes (Mano and Nemoto, 2012), including β-oxidation of IBA. Although IBA is a relatively abundant auxin, this was originally thought to be a minor biosynthetic route. However, the physiological importance of IBA-derived IAA has been demonstrated conclusively in *Arabidopsis* by elegant genetic analysis (Strader and Bartel, 2011; Strader *et al.*, 2011). IBA has also been proposed to be a hormone in its own right (Tognetti *et al.*, 2010). Very little is known regarding the functions of IBA in barley, but the data presented here demonstrate a role for IBA-derived IAA in root growth.

### HvABCD1 and HvABCD2 may differ in substrate specificity

Since CTS is a broad specificity transporter which plays several distinct roles in growth and development of *Arabidopsis*, the presence of two transporters in cereals is intriguing. Classically, gene duplication is considered to be a mechanism which increases expression diversity and permits the evolution of tissue or developmental specialization. Duplicate genes also play a role in the establishment of new functions or in subfunctionalization (Li *et al.*, 2005). The ABC transporter superfamily exhibits a particularly high level of gene birth and death, often associated with the acquisition of taxon-specific functions (Annilo *et al.*, 2006; Verrier *et al.*, 2008). *HvABCD1* and *HvABCD2* display similar expression patterns in embryo and aleurone, suggesting that they may play distinct roles in imbibed grains and indeed throughout the plant, since they are widely expressed in different tissues. This is consistent with their differential ability to complement *cts-1*. Whilst *HvABCD2* complemented all phenotypes tested, *HvACBD1* was apparently unable to mediate sufficient fatty acid transport to support seedling establishment when expressed in *Arabidopsis* and was unable to restore germination to *cts-1* seeds (Fig. 5D), suggesting differences in substrate specificity. Although, as discussed above, complementation depends on expression in the correct subcellular location at an appropriate level, it is clear that *HvACBD1* was expressed (and presumably targeted correctly) in the present experiments, since it conferred sensitivity of *cts-1* hypocotyls to high concentrations of IBA and partially complemented the seed size phenotype (Fig. 5). Transport studies will be required to determine unequivocally the substrate specificities of HvABCD1 and HvABCD2, and further analysis of RNAi lines may reveal additional, perhaps cereal-specific functions for these proteins. In conclusion, monocot and dicot ABCD transporters share a core set of functions, but the retention of two ‘full-length’ *HvABCD* genes in the grass lineage points to an important functional or regulatory diversification which awaits further investigation.

## Supplementary data

Supplementary data are available at *JXB* online.

Figure S1. Alignment of *HvABCD1* and *HvABCD2* cDNA sequences, showing the region used for the RNA interference construct.

Figure S2. Expression analysis of *HvABCD1* and *HvABCD2.*

Figure S3. Germination of RNAi lines and corresponding nulls over 7 d.

Figure. S4. Q-PCR analysis of gene expression in *Arabidopsis* transgenic lines and controls.

Table S1. Primers used in this study.

Supplementary Data

## Abbreviations:

ABC transporter: ATP-binding cassette transporter
ABCD: ATP-binding cassette transporter, subclass D
CTS: COMATOSE
2,4-DB: 2,4-dichlorophenoxybutyrate
GA: gibberellic acid
IAA: indole acetic acid
IBA: indole butyric acid
JA: jasmonic acid
NBD: nucleotide-binding domain
OPDA: 12-oxophytodienoic acid
TMD: transmembrane domain.
