# Supplementary material for: Barley has two peroxisomal ABC transporters with multiple functions in β-oxidation
Source: J Exp Bot. 2014 Jun 9;65(17):4833–47. doi: 10.1093/jxb/eru243 (PMC4144768; doi:10.1093/jxb/eru243)
Supplement: Supplementary Data [file supp_65_17_4833__index.html]

Barley has two peroxisomal ABC transporters with multiple functions in β-oxidation — Barley has two peroxisomal ABC transporters with multiple functions in β-oxidation — Supplementary Data 

# Barley has two peroxisomal ABC transporters with multiple functions in β-oxidation

## Supplementary Data

Data files

**Files in this Data Supplement:**

- Supplementary Data - Supplementary Data
