# Supplementary material for: Barley has two peroxisomal ABC transporters with multiple functions in β-oxidation
Source: J Exp Bot. 2014 Jun 9;65(17):4833–47. doi: 10.1093/jxb/eru243 (PMC4144768; doi:10.1093/jxb/eru243)
Supplement: Supplementary Data [file supp_eru243_jexbot123471_file001.pdf]

Figure S1. Alignment of *HvABCD1* and *HvABCD2* cDNA sequences, showing the region used for the RNA interference construct

The alignment was created using ClustalW2 multiple sequence alignment.

```
HvABCD2         ATGTCAGGTACCATGCCGTCCCTGCAGCTGCTGCAGCTCACCGAGCGTGGCCGCGGGCTG 60
RNAi-insert     ------------------------------------------------------------
HvABCD1         ------------ATGTCATCTCTTCAGTTATTGCAACTAACAGAGCATGGGCGGAACCTT 48


HvABCD2         CTCGCGTCCCGGAGGAGGACGCTGGCGGTTGTCTCGGGCGCGCTGGTCGCTGGCGGGGCG 120
RNAi-insert     ------------------------------------------------------------
HvABCD1         TTTTCTTCGAGAAGGAGGACGCTTGCAGTTGTTTCTGGTGCACTGCTTGCTGGTGGAACT 108


HvABCD2         TTGGCGTACGCGCGGTCGGGCCGAGGCCAAAGGAGGCGAGGCCGTCCGGAGGCCGCCGCC 180
RNAi-insert     ------------------------------------------------------------
HvABCD1         TTAGCATACGCGCAGACAAGTCGACG---------GAGGAAACATCGGGAAGAAAATCCT 159


HvABCD2         GCCAATGATGGCGGCGATGCGTTGGGCAGGAATGGGGAGAGGCTGGGTCACAGCGGCACG 240
RNAi-insert     ------------------------------------------------------------
HvABCD1         TGTAATGATG-CAAATGTACATAG--CAGGAGTGAAGAGAACATTAGTCAAAACGGTGTC 216


HvABCD2         GATGGCCGGTTGGCCGGGACGACGAAGCGGAGGAAGAGCGCGCTGAAGTCCTTGCATTTC 300
RNAi-insert     ------------------------------------------------------------
HvABCD1         GATGGTAAAGTG---GTAAAAACAAGGAAAAAGAAAAATTTATTGAAATCCTTACATTTC 273


HvABCD2         TTGGCCGCTATCCTGCTCAAGAAGATTGGACCGAGTGGCACGCGATACCTTCTTGGCTTG 360
RNAi-insert     ------------------------------------------------------------
HvABCD1         CTGGCTGCTATTTTACTTAAGAAGATTGGTCCAAGCGGAACAAATTACCTTCTTGGCTTG 333


HvABCD2         ATGTTGACAGCCGTGCTGCGTACTGCTGTCGGGCATAGATTAGCAAAAGTTCAAGGCTTT 420
RNAi-insert     ------------------------------------------------------------
HvABCD1         ATGTTAACAGCAGTGTTACGTACAACCATTGGTCACAGATTAGCAAAAGTTCAAGGGTAT 393


HvABCD2         CTATTTAAAGCGGCATTTCTTCGGCGTGTTCCAACCTTTACGCGCCTAATTATAGAGAAT 480
RNAi-insert     ------------------------------------------------------------
HvABCD1         TTGTTTAAATCCGCGTTTCTTCGGCGTGTTCCGACTTTCACACGCCTAATCATTGAAAAT 453


HvABCD2         CTGATCTTATGCTTTCTCCAATCAGCAGTATACCAGACCTCAAAATACTTAACTGGGTCC 540
RNAi-insert     ------------------------------------------------------------
HvABCD1         CTTCTATTATGCTTTCTTCAGTCCACGGTATATCAGACCTCAAAGTACTTGACAGGATCC 513


HvABCD2         TTGAACTTGCGCTTCAAGAAAATTTTAACAGACCTCGTCCATGCCGATTATTTCCAGAAT 600
RNAi-insert     ------------------------------------------------------------
HvABCD1         TTAAGCTTGCGCTTCAAGAAAATTTTGACGGATCTCATCCATGCTGATTACTTCGAGAAT 573


HvABCD2         ATGGTTTACTACAAGATCTCTCATGTGGATCATCGAATATCAAATCCGGAGCAAAGGATT 660
RNAi-insert     ------------------------------------------------------------
HvABCD1         ATGGTTTACTACAAGATCTCACATGTAGATCATCGGATTTCAAACCCAGAGCAAAGGATT 633


HvABCD2         GCCAGTGATATTCCAAAGTTCTCTTCTGAACTTAGTGAACTTGTACAGGATGATTTGGCT 720
RNAi-insert     ------------------------------------------------------------
HvABCD1         GCTAGTGATATTCCTAAGTTCTGCGCAGGACTAAGTGACCTTGTACAAGATGATCTGATT 693


HvABCD2         GCAGTTGCAGAAGGGTTGATATATACATGGCGTCTCTGTTCTTATGCTAGTCCGAAATAC 780
RNAi-insert     ------------------------------------------------------------
HvABCD1         GCAGTTGCAGATGGGTTAATATACATCTGGCGCATCTGCTCTTATGCAAGTCCGAAATAT 753


HvABCD2         ATGCTTTGGATTCTGGCATATATACTAGTTGCTGGTGGAGCAATTAGAAATTTCTCTCCT 840
RNAi-insert     ------------------------------------------------------------
HvABCD1         GTTCTCTGGATTCTGGCATATGTACTTGGTGCTGGTGGCACAATTAGAAAATTTTCTCCT 813


HvABCD2         GCTTTTGGGAAGATGAAATCCACAGAGCAGCACTTAGAAGGGGAGTATCGTCAACTTCAT 900
RNAi-insert     ------------------------------------------------------------
HvABCD1         TCTTTTGGGAAGTTGAAAGCCATGGAACAACAACTAGAAGGGGAATATCGCCAGGTTCAT 873


HvABCD2         TCACGCTTAAGAACTCATGCTGAGAGTGTAGCATTTTATGGTGGTGAGAAGAGAGAAGAA 960
RNAi-insert     ------------------------------------------------------------
HvABCD1         TCACGACTGAGAACTCATGCTGAGAGCGTGGCATTTTATGGTGGTGAGAACAGAGAAGAA 933
```

```
HvABCD2         TATCATATTATGCAGCGATTTCGAGCTCTTGTTGGGCACTTGAAGCATGTACTCCATGAA 1020
RNAi-insert     ------------------------------------------------------------
HvABCD1         TCACACATTATGCAGCGGTTCCAGGCTCTTGTGCGGCACTTGAATGTTGTTCTTCATGAG 993

HvABCD2         AACTGGTGGTTCGGCATGATTCAAGATTTCTTTCTGAAGTACTTTGGGGCCACTGTAGCA 1080
RNAi-insert     ------------------------------------------------------------
HvABCD1         AACTGGTGGTTTGGCATGATTCAAGATTTTCTTCTGAAGTATCTTGGTGCCACAGTGGGA 1053

HvABCD2         GTTGTCCTTATTATCGAACCGTTCTTCTCCGGTGACCTTAGACCTGATTCATCTACCATA 1140
RNAi-insert     ------------------------------------------------------------
HvABCD1         GTTATCTTGATTGTCGAACCTTTCTTTGCGGGAGATCTTAAACCTGATACATCTACTTTA 1113

HvABCD2         GGAAGGGCTGATATGTTGAGTAACCTTCGATACCACACTAGCGTGATCATAGCACTATTT 1200
RNAi-insert     ------------------------------------------------------------
HvABCD1         GGGCGGGCAGAGATGTTGAGCAATCTTAGATATCACACAAGCGTGATAATATCATTATTC 1173

HvABCD2         CAGTCTCTTGGGACCCTCTCTATCAGCTCTAGGCGTTTGAATATCCTGAGTGGCTATGCA 1260
RNAi-insert     ------------------------------------------------------------
HvABCD1         CAGTCGCTTGGCACCCTTTCTACCAGCTCAAGACGTTTAAATCTTCTCAGTGGCTATGCA 1233

HvABCD2         GACCGGATTCGTGAGTTACTAGATGTTTCACGTGAGCTGTCTGGGGTCCGTGATAGATCG 1320
RNAi-insert     ------------------------------------------------------------
HvABCD1         GACCGTATTCACGAGTTACTGGATGTTTCACGTGAGCTATCTGGGGTTCGCGACAGGTCG 1293

HvABCD2         TTGAATCACAGCTCTTCTGTTGGGAATTACATCAGCGAAGCAAACCATATAGAATTTTCT 1380
RNAi-insert     ------------------------------------------------------------
HvABCD1         ATGAGTCGAAATTCCTCTGCCAAAAACTATATTAGTGAGGCAAACTATATAGAATTTTCA 1353

HvABCD2         GGTGTTAAGGTGGTGACACCCGCTGGGAACGTTTTGGTTGACGATTTAACTCTCCGAGTA 1440
RNAi-insert     ------------------------------------------------------------
HvABCD1         GGTGTCAAGGTGGTGACACCCTCTGGGAATGTCTTGGTTGATGATTTAACTCTCCGGGTG 1413

HvABCD2         GAAACTGGATCTAATCTTTTAATCACCGGTCCCAATGGTAGTGGAAAAAGCTCCCTTTTT 1500
RNAi-insert     ------------------------------------------------------------
HvABCD1         GAGTCGGGTTCTAATCTTTTGATCACTGGTCCCAACGGTAGTGGAAAAAGCTCTCTTTTC 1473

HvABCD2         CGGGTTCTTGGAGGCCTATGGCCGCTAGTATCTGGCCATATTGTCAAACCTGGTGTTGGT 1560
RNAi-insert     ------------------------------------------------------------
HvABCD1         CGTGTTCTTGGGGGTCTGTGGCCACTTGTATCTGGCCACATTGTCAAACCTGGTGTTGGT 1533

HvABCD2         TCCGATCTTAACAAGGAAATCTTTTATGTCCCACAAAGACCATACACAGCTGTTGGCACA 1620
RNAi-insert     ------------------------------------------------------------
HvABCD1         TCTAATCTTAACAAGGAAATATTTTATGTCCCCCAGCGACCATATACAGCTGTTGGAACA 1593

HvABCD2         CTTCGCGAACAACTAATCTATCCTCTTACAGCAGATCAGGAGACTGAACCACTTACCTAT 1680
RNAi-insert     ------------------------------------------------------------
HvABCD1         CTGCGCGACCAGTTAATCTATCCACTTACAGCAGATCAGGAAACCGAACCACTTAGCTAT 1653

HvABCD2         GATGGCATGGTGGACCTTCTAAAGAATGTTGATCTGGAATACTTGCTAGAACGTTATCCT 1740
RNAi-insert     ------------------------------------------------------------
HvABCD1         GGTGGTATGGTGGATCTTCTAAAGAATGTTGATTTAGAATACTTGCTAGAACGCTACCCT 1713

HvABCD2         CTTGACAAGGAAGTTAACTGGGGTGATGAGCTATCCCTTGGCGAGCAACAAAGATTAGGA 1800
RNAi-insert     ------------------------------------------------------------
HvABCD1         GTCGACAAGGAAGTTAACTGGGGCGATGAGTTGTCTCTTGGTGAGCAACAGAGATTGGGA 1773

HvABCD2         ATGGCCAGGCTGTTCTACCATAAGCCAAAATTTGCTATTCTTGATGAGTGTACCAGTGCT 1860
RNAi-insert     ------------------------------------------------------------
HvABCD1         ATGGCCAGATTGTTCTACCATAAGCCCAAGTTTGCCATCCTGGATGAGTGTACTAGTGCT 1833

HvABCD2         GTGACAATTGATATGGAAGAACGTTTTTGCAAAAAGGTTCGAGCGATGGGAACATCATGC 1920
RNAi-insert     ------------------------------------------------------------
HvABCD1         GTGACGACTGATATGGAAGAACGTTTCTGCAATAGGGTCCGAGCAATGGGCACGTCATGC 1893

HvABCD2         ATAACAATATCTCATCGCCCAGCATTAGTTGCATTTCATAATATTGTTTTGTCATTGGAC 1980
RNAi-insert     ------------------------------------------------------------
HvABCD1         ATAACAATATCTCACCGACCAGCATTAGTTGCTTTTCATGATGTCGTTTTGTCCTTGGAT 1953
```

```
HvABCD2         GGTGAAGGAGGGTGGGATGTTCAGCACAGAAGGGATGACTCATCCTTTTACACTGAA--- 2037
RNAi-insert     ------------------------------------------------------------
HvABCD1         GGTGAAGGAGGATGGAAAGTTCAAGATAACAGAAATGGCTCCTTCCTTCCTACTGAATCG 2013

HvABCD2         GACTCTG---AACTTACATCACTGGAAACTGAACGTAAATCGGATGCCCTAATTGTTCAG 2094
RNAi-insert     ------------------------------------------------------------
HvABCD1         GAGTTTGGTGCATTGAAGTCATCAGAAACTGACCGCAAATCTGACGCACTTGCTGTTCAA 2073

HvABCD2         AGGGCCTTTATGAACAGGGCAAAGAGCAATGCTTCATTAAGGTCCAAG---GATTCCTAC 2151
RNAi-insert     ------------------------------------------------------------
HvABCD1         AGGGCTTTCAGTGCTAATACAAAGGAAAATACTTTATCAGGCCCCAAGGACCATTCTTAT 2133

HvABCD2         TCTACAAAGGTCATAGCAAATTCCGCCAAGTTGGAAACAGGACAAACAGTACGAACACCT 2211
RNAi-insert     ------------------------------------------------------------
HvABCD1         TCAACACAGGTCATAGCCACTTCCCCCAATATGGAAATAGAATCCACAGAACAACCACAT 2193

HvABCD2         GTAATCCCACATTTACAATGCTCCCCAAGGCCTCTGCCTCTGAGAGCGGCTGCAATGCTC 2271
RNAi-insert     ------------------------------------------------------------
HvABCD1         CTTATCCCACAATTGCAGTGCTCGCCGAGACCTTTGCCTGTTAGAGTTGCTGCAATGTCT 2253

HvABCD2         AAAATATTGATTCCTAAATTGCTTGACAAACAAGGAGGGCAGTTGCTTGCTGTCGCTCTA 2331
RNAi-insert     ------------------------------------------------------------
HvABCD1         AAAATACTGGTTCCTAAAGTCATTGATAAGCAAGGGGCACAATTACTTGCAGTTGCACTA 2313

HvABCD2         CTTATTTTCTCTCGCACTGTGATCTCGGATCGTATTGCTTCGCTGAATGGGACCACTGTG 2391
RNAi-insert     ------------------------------------------------------------
HvABCD1         CTAGTACTATCGCGGACTTGGATATCTGATCGTATAGCTTCACTGAACGGGACTAGTGTT 2373

HvABCD2         AAGTTTGTCTTGGAGCAGGATAAAGCTGCTTTTGTTCGGCTGGTTGGCGTCAGTGTTCTG 2451
RNAi-insert     ------------------------------------------------------------
HvABCD1         AAGTATGTCCTGGAGCAGGACAAGGCTGCCTTCATTCGATTGATCGGGACCAGTGTAATG 2433

HvABCD2         CAGAGTGCTGCAAATTCTTTTGTCGCACCATCTCTGAGAACCCTTACTTCAAGACTTGCC 2511
RNAi-insert     ------------------------------------------------------------
HvABCD1         CAAAGTGCTGCAAATTCAATTGTGGCACCATCACTGAGACACCTTACTTCAAAGATTGCT 2493

HvABCD2         CTTGGATGGCGAATTCGCTTGACCAACCATTTGCTTCAATATTATTTGAGAAGAAATGCA 2571
RNAi-insert     ------------------------------------------------------------
HvABCD1         CTTGGATGGCGAATTCGCATGACAAACCATCTACTTGCATATTATTTGAAAAGAAATGCT 2553

HvABCD2         TTCTACAAGGTATTCAATATGTCGGGTAAAAGTATAGATGCAGACCAAAGACTAACACTT 2631
RNAi-insert     ------------------------------------------------------------
HvABCD1         TTTTACAAGGTATTTAATATGACAGGCACAGATATTGATGCAGACCAAAGAATAACACGT 2613

HvABCD2         GATGTGGATAAGTTGACCACTGACCTTGCTGGCTTGGTTACTGGAATGGTAAAACCACTA 2691
RNAi-insert     ------------------------------------------------------------
HvABCD1         GATGTGGAGAAGTTGACCAATGATCTTGCTGGCTTAGTTACTGGAATGGTGAAACCATCA 2673

HvABCD2         GTTGACATTCTTTGGTTTACATGGAGAATGAAGCTTTTGTCCGGCCGTAGAGGAGTTGGT 2751
RNAi-insert     ------------------------------------------------------------
HvABCD1         GTTGACATTCTTTGGTTTACATGGAGAATGAAGCTTTTATCTGGGAGAAGAGGAGTTGCT 2733

HvABCD2         ATATTGTATGCTTACATGTTACTAGGATTGGGTTTCCTGAGAGCTATATCCCCTGACTTT 2811
RNAi-insert     ---------------------------------TTCCTGAGAGCTATATCCCCTGACTTT 27
HvABCD1         ATTTTGTACGCATACATGTTGCTAGGGCTTGGTTTTCTAAGAGCCGTTTCCCCTGATTTC 2793
                                                 ** **.***** .*:******** **

HvABCD2         GGTCATCTTGCAAGTGAGGAACAAGAACTTGAAGGAACATTCAGGTTTATGCACTCGAGG 2871
RNAi-insert     GGTCATCTTGCAAGTGAGGAACAAGAACTTGAAGGAACATTCAGGTTTATGCACTCGAGG 87
HvABCD1         GGCGATCTTTCAAACCAAGAACAAGAACTTGAAAGTTCTTTCAGGTTCATGCACTCAAGA 2853
                **  ***** ***.  *.***************.*::*:******** ********.**.

HvABCD2         CTGCGGACGCATGCTGAATCAATTGCTTTCTTTGGTGGTGGTTCAAGAGAAAAAGCTATG 2931
RNAi-insert     CTGCGGACGCATGCTGAATCAATTGCTTTCTTTGGTGGTGGTTCAAGAGAAAAAGCTATG 147
HvABCD1         CTGCGAACACATGCTGAGTCAATTGCTTTCTTTGGTGGTGGATCAAGGGAAAGAGCTATG 2913
                *****.**.********.**********************:*****.****.*******

HvABCD2         GTTGAGGCTAAATTCATGAAACTGCTTAACCACTCAAAGGTTCTGTTGAGAAAACAATGG 2991
RNAi-insert     GTTGAGGCTAAATTCATGAAACTGCTTAACCACTCAAAGGTTCTGTTGAGAAAACAATGG 207
HvABCD1         GTTGAAGCTAAGTTCGCAACTTTGCTTAACCACTCGAAGATTCTATTGAGGAAAAGATGG 2973
                *****.*****.***. .*.: *************.***.****.*****.***..****

HvABCD2         CTTTATGGTGTTGTTGATGATTTTGTGACAAAGCAACTCCCACACAATGTGACATGGGGT 3051
RNAi-insert     CTTTATGGTGTTGTTGATGATTTTGTGACAAAGCAACTCCCACACAATGTGACATGGGGT 267
```

```
HvABCD1            CTTTATGGTATTTTTGATGATTTCGTGACGAAGCAGCTGCCTCATAATGTGACGTGGGGA 3033
                   *********.** ********** *****.*****.** **:** ********.*****:

HvABCD2            CTGAGTTTGTTATATGCTCTGGAGCACAAGGGAGACAGAGCTTTGACTTCAACACAAGGA 3111
RNAi-insert        CTGAGTTTGTTATATGCTCTGGAGCACAAGGGAGACAGAGCTTTGACTTCAACACAAGGA 327
HvABCD1            TTGAGTTTGTTATATGCCTTGGAACACAAGGGGGACCGAGCTTTGACTTCAGCGCAAGGA 3093
                    ****************  ****.********.***.**************.*.******

HvABCD2            GAGCTGGCACATGCTCTGCGGTTCTTGGCATCAGTGGTGTCACAAAGCTTCATAGCTTTC 3171
RNAi-insert        GAGCTGGCACATGCTCTGCGGTTCTTGGCATCAGTGGTGTCACAAAGCTTCATAGCTTTC 387
HvABCD1            GAGTTAGCACATGCTCTGCGGTTCTTGGCATCTGTAGTGTCACAAAGTTTCATAGCTTTT 3153
                   *** *.**************************:**.*********** *********** 

HvABCD2            GGCGACATTCTTGAGTTACATAAGAAATTCCTTGAACTTTCTGGTGGGGTTAATCGGATC 3231
RNAi-insert        GGCGACATTCTTGAGTTACATAAGAAATTCCTTGAACTTTCTGGTGGGGTTAATCGGATC 447
HvABCD1            GGTGACATTCTCGAGTTGCATAAGAAATTCCTTGAACTTTCTGGTGGTGTCAATAGGATC 3213
                   ** ******** *****.***************************** ** ***.*****

HvABCD2            TTTGAACTCGAGGAGATTCTGCGTGTAGCTCAAAAGGATACCCCAGTGCCTTCCAGTGCC 3291
RNAi-insert        TTTGAACTCGAGGAGATTCTGCGTGTAGCTCAAAAGGATACCCCAGTGCC---------- 497
HvABCD1            TTTGAACTCGAGGAGCTTCTAAAGGCATCTCAAAGCAATGCTGTCATGCCATCAAATGTT 3273
                   ***************.****... * * ******. .**.*   ..****

HvABCD2            ATTAGTGCAGCCTCAGACGAAATTATTGAATTCCATGAAGTGGATATTGTAACACCATCC 3351
RNAi-insert        ------------------------------------------------------------
HvABCD1            ATCAGTGTGCCATCTGAACAAACTATTTCCTTCCGTGATGTGGATATTGTGACCCCATCG 3333

HvABCD2            CAGAAGCTATTAGCTAGGAAGCTGTCATGCAGTGTGGTACAAGGGAAAAGCCTTCTCTTG 3411
RNAi-insert        ------------------------------------------------------------
HvABCD1            CGAAAGCTACTGGCTAGCCAATTGTCTTGTGATGTGTCTCAAGGAAAAAGCCTTCTTGTC 3393

HvABCD2            ACTGGCCCCAATGGTACCGGGAAGAGCTCTGTTTTTAGGGTGCTCAGAGATTTATGGCCT 3471
RNAi-insert        ------------------------------------------------------------
HvABCD1            ACTGGTCCAAATGGCAGTGGCAAAAGTTCCATTTTCAGGGTGCTCCAAAGTTTGTGGCCT 3453

HvABCD2            GCCTTTTCTGGGAGAGTTACCAAGCCCTCTGAAGGGATGTTCCATGTTCCTCAGAGTCCA 3531
RNAi-insert        ------------------------------------------------------------
HvABCD1            GTAGCATCTGGAAGGCTCACTGTGCCATCTGAAGGGATATTTCACGTTCCTCAGCGTCCA 3513

HvABCD2            TACACCAGCCTGGGAACTCTTAGGGATCAGATCATATACCCTCTGTCAAGGGAGGAAGCA 3591
RNAi-insert        ------------------------------------------------------------
HvABCD1            TATACTTGCCTTGGAACCTTGAGGGATCAGATCATATATCCTCTCTCACGTGAGGAGGCA 3573

HvABCD2            GAGATGAAGATTCTTTCGTTATATAAGTCTAGCAACAGGTCTAGTGCCCCTGAGTTGCTA 3651
RNAi-insert        ------------------------------------------------------------
HvABCD1            GAGTTGAAGATGGTTACATTATCCAAAACAAGTGACAGGTCTA---CACCG------CTG 3624

HvABCD2            GACGATCACCTGAAGACGGTTCTTGTGAATGTTCGGTTGGTCTATCTTCTAGAGAGGGAA 3711
RNAi-insert        ------------------------------------------------------------
HvABCD1            GACGATCACCTGAGGACAATTCTAGAGAATGTTCGCTTGGTTTATCTTCTGGAAAGAGAA 3684

HvABCD2            GGCTGGGACTCTACCCCAAACTGGGAAGACGTTTTATCGCTGGGAGAACAGCAGAGGCTT 3771
RNAi-insert        ------------------------------------------------------------
HvABCD1            GGTTGGGATGCTACTCCCAATTGGGAAGACATTTTGTCCTTGGGAGAGCAGCAGAGGCTA 3744

HvABCD2            GGGATGGCTCGTTTGTTCTTCCACCATCCTAAATTCGGCATCCTCGACGAGTGCACAAAT 3831
RNAi-insert        ------------------------------------------------------------
HvABCD1            GGCATGGCTCGTCTGTTCTTTCACTCTCCTAAGTTTGGTATCCTCGACGAGTGCACCAAT 3804

HvABCD2            GCTACGAGTGTTGATGTTGAAGAGCATCTGTACAGGCTAGCAACAGACATGGGCATAACT 3891
RNAi-insert        ------------------------------------------------------------
HvABCD1            GCTACGAGTGTCGACGTGGAGGAGCATTTGTACAGGCTAGCAACCGACTTGGGTATAACT 3864

HvABCD2            GTGATCACTTCGTCACAAAGGCCTGCTCTTATACCCTTCCACTCCGTGGAGCTTAAACTC 3951
RNAi-insert        ------------------------------------------------------------
HvABCD1            GTGGTCACTTCCTCCCAAAGACCTGCTCTCATACCCTTCCATTCTTTGGAGCTCAAACTC 3924

HvABCD2            ATCGATGGTGAGGGGAACTGGGAGCTATGCGAGATCCAACATTGA 3996
RNAi-insert        ---------------------------------------------
HvABCD1            ATCGACGGTGAAGGAAAGTGGGAGCTGTGTTCAATCAACCACTAG 3969
```

A

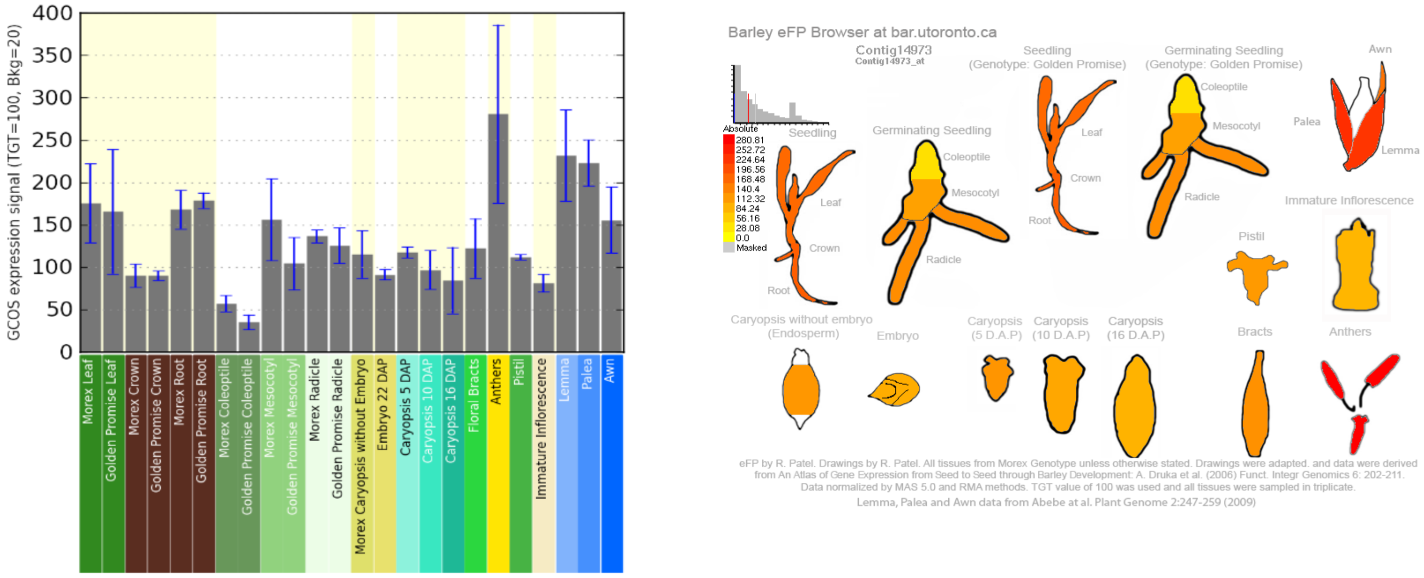

B

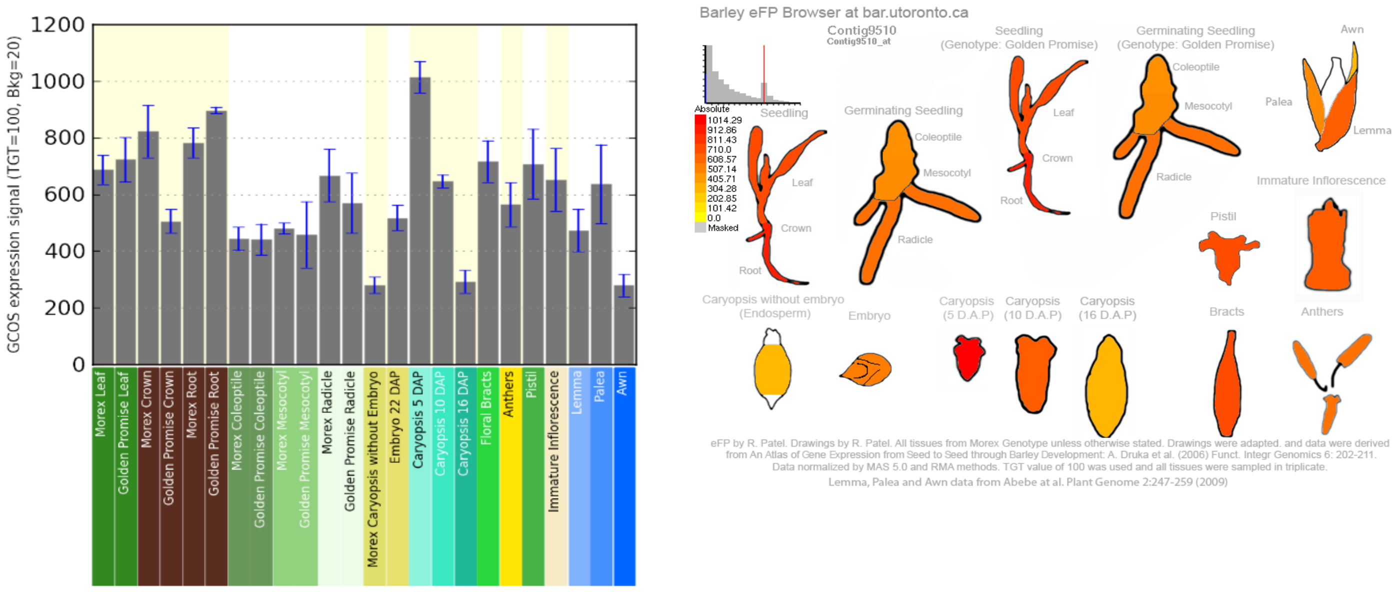

Figure S2. Expression analysis of *HvABCD1* and *2*

Histograms and and electronic fluorescent pictographs for (A) *HvABCD1* and (B) *HvABCD2* were obtained by interrogating the Bio-Array Resource barley eFP browser.

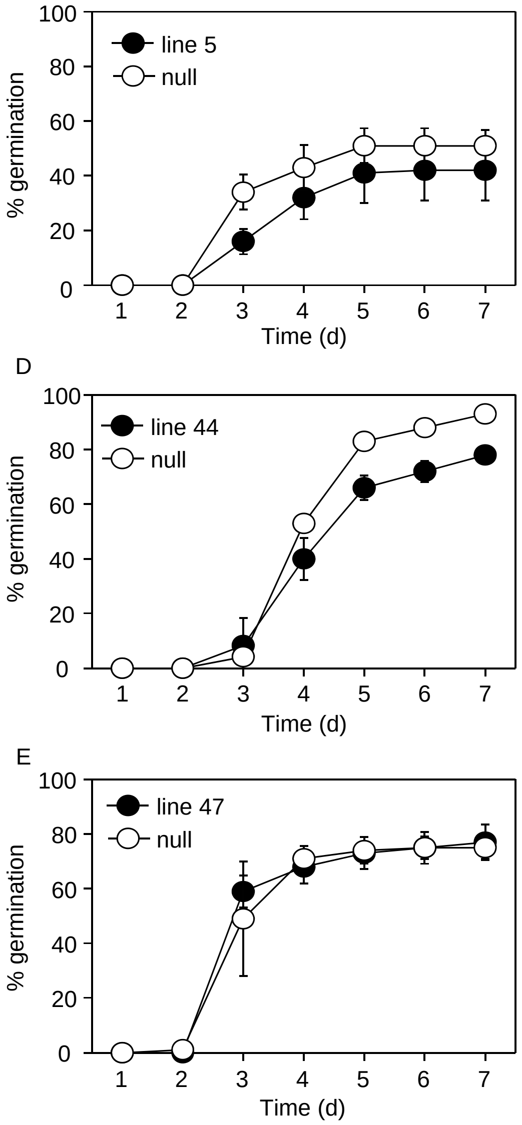

Figure S3. Germination of RNAi lines and corresponding nulls over 7 d.

Values are means ± SE (n=3; 50 seeds per replicate).

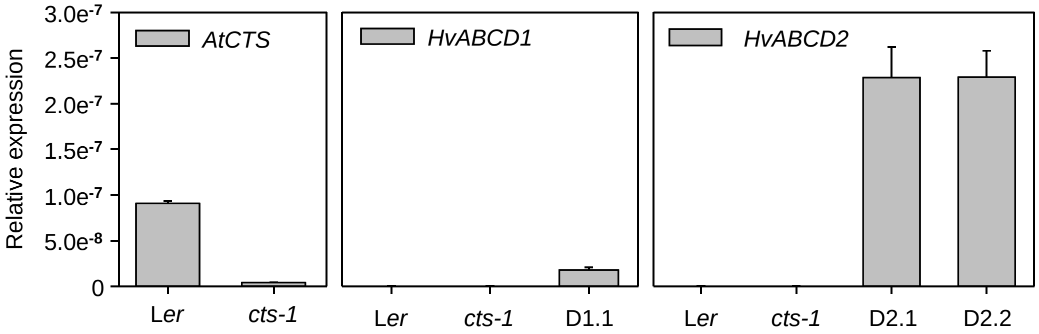

Figure. S4. Q-PCR analysis of gene expression in Arabidopsis transgenic lines and controls.

Transcript abundance is shown relative to housekeeping genes. RNA was isolated from seedlings grown on 0.5 x MS containing 0.5 % sucrose. The testae of *cts-1* and line A19 seeds were mechanically disrupted to induce germination. Values are means ± SE (n=3)

Supplementary Data

Supplementary Table 1 Primers used in this study

Name	Sequence (5'-3')
CTS-a start F	CAATGTCATCTCTTCAGTTATTGC
Hv14973 R2	GGTGCTTCCTCTGTTTGTTG
HvABCD1 (CTSa) F	AGTGTGCCATCTGAACAAAC
HvABCD1 (CTSa) R	AATGGAACTTTTGCCACTGC
HvABCD2 (CTSb) F	CAGCCTCTCCCCATTCCT
HvABCD2 (CTSb) R	AGTGCAGCCTCAGACGAAAT
α-tub F	AGTGTCCTGTCCACCCACTC
α-tub R	AGCATGAAGTGGATCCTTGG
PM19F	GGAGGCGTTCGTCATCATC
PM19R	TCCTATTTAGACCCTTGCCAC
HvABCD RNAi F	TTCCTGAGAGCTATATCCCCTGA
HvABCD RNAi R	GGCACTGGGGTATCCTTTTGAG
HvELF1F	CCTGCCCAAGTTCCTCAA
HvELF1R	GGCTTGGTGGGAATCATCT
HvTubulinF	ACACCATTGGCAAGGAGATT
HvTubulinR	TGTCGGACAGCTTCCTGATAC
HvJIP2 F	ACCTTCATGACGGCAACTG
HvJIP2 R	CTCCCGGTGTTCTTTTCATC
HvJRG1.2 F	GAAATGGAGGGGGTGATAAAG
HvJRG1.2 R	CGCTGCTAACAGTGATGCTC
AtCTS F	GAGATTAGGCATGGCACGTT
AtCTS R	GTCGCATTTGTGCATTCATC
AtCTL1 F	AGTGGAGAGGCTGCAGAAGA
AtCTL1 R	CTCGGGTAGCACGAGCTTTA
AtCTL3 F	GAAGTGTCTCGACAAAGGTCGT
AtCTL3 R	CCTTTTGGCACTTCTGGTG
AtCTSp5'Nco	TAACCATGGACTTGGAAGAAGGCGGTGA
AtCTSp3'Kpn	TAAGGTACCTGACATGGCCTGGAATTGAGTATA
HvCTSa Kpn5'	TTAGGTACCATGTCATCTCTTCAGTTATTGCAA
HvCTSa Not3'	TTTGCGGCCGCTGGTGCTTCCTCTGTTTGTTG
CTSb Kpn5'	TTAGGTACCATGCCGTCCCTGCAGCTG
CTSb Not I-Bgl II R	TTAGCGGCCGCATCACTGGCAATCCTTTGCTC
CTSb Bgl IIF	ACTGCTGTCGGGCATAGATT
CTSb Not3'	ATTGCGGCCGCCGACCCGGTATGAACAACTT
FT206	CCCAATGGTACCAAAATGTCATCTCTTCAG
FT207	CGAAGGCTGCAGTCAAGCGTAATCTGGAACATCGTATGGGTAGTGGTTG ATTGAACAC
FT208	CCCAATGGATCCAAAATGCCGTCCCTGCAGC
FT209	CGAAGGGTCGACTCAAGCGTAATCTGGAACATCGTATGGGTAATGTTGG

	ATCTCGCATAGC

Barley accession numbers and Arabidopsis gene identifiers: HvJIP2, CAA47017; HvJIP37, CAA58110; HvJRG1.2, AAB72097; AtCTS, AT4G39850; AtCTL1, At1g04850; AtCTL3, At5g18800.
